# Deep learning-enabled exploration of global spectral features for photosynthetic capacity estimation

**DOI:** 10.3389/fpls.2024.1499875

**Published:** 2025-01-13

**Authors:** Xianzhi Deng, Xiaolong Hu, Liangsheng Shi, Chenye Su, Jinmin Li, Shuai Du, Shenji Li

**Affiliations:** ^1^ State Key Laboratory of Water Resources Engineering And Management, Wuhan University, Wuhan, Hubei, China; ^2^ Urban Operation Management Center of Hengsha Township, Shanghai, China

**Keywords:** hyperspectral data, spectral sensitive band, vegetation index, photosynthetic capacity, deep learning, power compression

## Abstract

Spectral analysis is a widely used method for monitoring photosynthetic capacity. However, vegetation indices-based linear regression exhibits insufficient utilization of spectral information, while full spectra-based traditional machine learning has limited representational capacity (partial least squares regression) or uninterpretable (convolution). In this study, we proposed a deep learning model with enhanced interpretability based on attention and vegetation indices calculation for global spectral feature mining to accurately estimate photosynthetic capacity. We explored the ability of the model to uncover the optimal vegetation indices form and illustrated its advantage over traditional methods. Furthermore, we verified that power compression was an effective method for spectral processing. Our results demonstrated that the new model outperformed traditional models, with an increase in the coefficient of determination (R^2^) of 0.01-0.43 and a decrease in root mean square error (RMSE) of 1.58-12.48 μmol m^-2^ s^-1^. The best performance of our model in R^2^ was 0.86 and 0.81 for maximum carboxylation rate (*V_cmax_*) and maximum electron transport rate (*J_max_*), respectively. The photosynthesis-sensitive spectral bands identified by our model were predominantly in the visible range. The most sensitive vegetation indices form discovered by our model was $\frac{Reflectance_{near-infrared}+Reflectance_{green/blue}}{Reflectance_{near-infrared}\times Reflectance_{red}}$ . Our model provides a new framework for interpreting spectral information and accurately estimating photosynthetic capacity.

## 1 Introduction

Photosynthesis plays a critical role in the carbon uptake of vegetation and significantly impacts food production (Friedlingstein et al., 2022). The capacity of photosynthesis in C_3_ crops relies on two critical physiological parameters: the maximum carboxylation rate (*V_cmax_*) and the maximum electron transport rate (*J_max_*) (Long and Bernacchi, 2003). Accurate estimation of these biochemical parameters that determined by modelling CO_2_ assimilation rate versus intercellular CO_2_ concentration (*A-C_i_*) curves is important for describing the complex dynamics of photosynthetic performance in various crops (Farquhar et al., 1980; Van der Tol et al., 2009; Zhang et al., 2014).

The *V_cmax_* and *J_max_* is typically measured via gas exchange systems, which is expensive and time-consuming. Due to the intrinsic mechanisms of reflectance spectroscopy in response to photosynthetic physiological processes, the excellent efficacy of spectral reflectance in accurately estimating *V_cmax_* and *J_max_* has been widely proved across different species and temperature ranges (Serbin et al., 2012, 2015; Heckmann et al., 2017; Silva-Perez et al., 2018; Kumagai et al., 2022).

Previous studies have demonstrated that specific wavelengths are highly sensitive to photosynthetic traits, making them valuable for estimating photosynthetic capacity. Light absorbed by chlorophyll pigments, especially in the blue and red regions (400-700 nm), drives key photosynthetic processes like electron flow and carbon fixation (Gitelson et al., 2022). While green light (500-570 nm) is less absorbed, it still plays a role in overall photosynthetic efficiency, which has been shown to be comparable to that of red light (Wolf and Blankenship, 2019; Gitelson et al., 2022). Far-red light (700-750 nm), although not directly involved in oxygen release, contributes by stimulating cyclic electron flow in Photosystem II (PSII) and Photosystem I (PSI), which enhances the overall efficiency of photosynthesis (Kramer and Sacksteder, 1998; Cruz et al., 2001; Joliot and Joliot, 2005, 2006; Laisk et al., 2010). Furthermore, near-infrared (750-1200 nm) reflectance is primarily influenced by leaf structure and mesophyll cell characteristics, which are linked to photosynthetic performance (Terashima and Saeki, 1983). The mechanical link between spectra and photosynthesis provides the foundation for exploring the spectral features of photosynthetic capacity.

In recent years, advances in sensor-enabled photosynthetic measurements have shifted the research focus towards mining rich spectral information (Araus and Cairns, 2014). However, the limited availability of real-world measurements of photosynthetic data poses a challenge in mining hyperspectral data, especially when the sample size is smaller than the dimension of the hyperspectral data (Prasad and Bruce, 2008; Mojaradi et al., 2009; Bioucas-Dias et al., 2013). Additionally, the spectral reflectance captured by hyperspectral sensors is influenced by multiple factors, including the geometric structure of plants (Slaton et al., 2001) and leaf scattering characteristics (Grant, 1987). Accordingly, spurious spectral variations will be introduced in the recorded signals, blurring spectral signatures associated with target photosynthetic traits (Fu et al., 2020). To address these challenges posed by high dimensionality of hyperspectral data and complex biophysical mechanism in the response of spectral reflectance to photosynthesis, current studies propose two mainstream solutions: vegetation indices-based model and full spectra-based model.

The vegetation indices are constructed based on sensitive wavelength bands. The mechanical relationship between the photosynthetic traits and some specific sensitive wavelengths has been widely proved (Barnes et al., 2017). Previous studies revealed that the visible to near-infrared (VNIR: 400-1400 nm) region is essential for predicting *V_cmax_* and *J_max_* (Serbin et al., 2012; Barnes et al., 2017; Meacham-Hensold et al., 2019). The key wavelengths, including blue band at 450 nm (Meacham-Hensold et al., 2019), green band at 550 nm (Wang et al., 2021a), red band at 660 nm (Fu et al., 2020) and 680 nm (Meacham-Hensold et al., 2019), far red band at 700-720 nm (Fu et al., 2020; Wang et al., 2021a), and near-infrared region of 800-1400 nm (Serbin et al., 2012, 2015), are detected. Accordingly, the vegetation indices defined based on abovementioned wavelengths are widely used to estimate *V_cmax_* and *J_max_*, such as normalized difference vegetation index (NDVI), enhanced vegetation index (EVI), and ratio vegetation index (SR) (Fu et al., 2020; Camino et al., 2022; Guo et al., 2023; Song et al., 2023). Specifically, the photochemical reflectance index (PRI), which is defined at 531 and 570 nm wavelengths and indicates xanthophyll pigments, shows good performance for describing photosynthetic efficiency (Ainsworth et al., 2014; Barnes et al., 2017; Fu et al., 2022). The Structure Insensitive Pigment Index (SIPI), which is calculated using 445, 680 and 800 nm wavelengths and is sensitive to chlorophyll a and carotenoids, is also proved as a good proxy for photosynthetic traits (Wu et al., 2019; Fu et al., 2020; Yan et al., 2021). However, the single vegetation index fails to fully utilize hyperspectral information. Different forms of indices can yield varying results in estimating photosynthetic capacity (Jin et al., 2012). Finding an appropriate combination of sensitive bands and index forms becomes a challenging task for target traits estimation (Wu et al., 2008; Yao et al., 2010; Chen et al., 2022).

The full spectral profiles contain more abundant information compared to vegetation indices. Many studies directly use the hundreds of bands to quantify the photosynthetic traits (Serbin et al., 2012, 2015; Yendrek et al., 2017; Meacham-Hensold et al., 2020; Sexton et al., 2021). The popular method is to construct the statistical relationship between full spectral reflectance and photosynthetic parameters. Some classical machine learning algorithms including partial least squares regression (PLSR) (Meacham-Hensold et al., 2019; Fu et al., 2024b) and lasso regression (Pellikka et al., 2023), deep learning models including artificial neural network regression (Fu et al., 2019) and one dimensional convolutional neural network (OneDCNN) (Furbank et al., 2021; Fu et al., 2024a) have been employed to build the statistical model and show good performance. Moreover, deep learning methods demonstrate higher performance compared to classical machine learning approaches (Furbank et al., 2021; Deng et al., 2024). However, the full spectral-based deep learning model such as OneDCNN is highly likely to learn the spurious relationship and even distort our understanding of the true biophysical response due to lack of prior knowledge constraints, resulting in the poor generalization ability (Fu et al., 2022). Therefore, it is important to incorporate prior knowledge constraints on biophysical spectral response characteristics into the deep learning models to enhance their interpretability and generalization.

The effective means to address current issues in estimating photosynthetic parameters lies in the integration of methods that automatically mine multiple spectral bands and incorporate biophysical priors within deep learning models. Accurately estimating photosynthetic parameters requires identifying key bands and spectral features tied to biophysical characteristics. Recent advancements have introduced attention mechanisms as an effective method for selecting sensitive bands (Lorenzo et al., 2020; Zheng et al., 2022). Attention mechanisms enable the model to assign greater weight to regions of interest, thereby improving the selection of important features (Vaswani et al., 2017). Global attention approach provides a promising method for sensitive bands selection. The gumbel softmax, characterized by the output in the form of one-hot vectors, exhibits excellent performance in models with latent categorical variables (Jang et al., 2017). Incorporating gumbel softmax into attention mechanism for identifying sensitive bands shows great potential. While these methods offer improvements, the application of knowledge-guided deep learning for photosynthetic trait estimation is still relatively underexplored.

Traditional spectral analysis methods often rely on predefined sets of spectral bands and fixed vegetation indices, which can fail to capture the full complexity of spectral information or adapt to variations in spectral characteristics (Wu et al., 2008; Yao et al., 2010; Chen et al., 2022). These methods tend to be limited by the assumption that fixed spectral bands are sufficient for accurate parameter estimation, which often does not align well with the complex biophysical processes being studied. Knowledge-guided deep learning, which integrates physical constraints, network architecture design based on prior knowledge, and data preprocessing methods informed by biophysical principles, offers a promising alternative (De Bézenac et al., 2019; Yuan et al., 2020; Chen et al., 2021; Sridharan and Mota, 2023). However, research on the application of knowledge-guided deep learning for photosynthetic trait estimation remains limited. Our previous study pioneeringly proposed the SA-IndiceCNN model which integrates prior knowledge by designing vegetation indices calculation (Deng et al., 2024). However, this model feeds abstract features derived from large spectral band regions through dilated convolutions and pooling operations, which sacrifices the detailed information from individual bands and distorts the spectral band positions.

To overcome this limitation, we focus on sensitive spectral bands and their correct positioning, which is expected to provide more reliable biophysical priors and further improve performance of the knowledge-guided deep learning model for *V_cmax_*, and *J_max_* estimation. Unlike previous models that employed a single index form, we expect the most important index form could be automatically identified by gating mechanism (Yu et al., 2019). Additionally, appropriate spectral signal preprocessing is crucial for improving photosynthetic capacity estimation (Guo et al., 2023; Song et al., 2023). Power compression, a technique widely applied in deep learning for speech spectrum signal processing (Li et al., 2021b), can reduce dynamic range and balance the loss gap between different spectral regions, allowing the neural network to capture more detailed information in weak signal areas (Li et al., 2021b).

In this study, we propose a novel approach that combines global attention mechanisms and gumbel softmax to identify sensitive spectral bands, addressing the limitations of traditional methods in spectral information utilization. A specialized loss function is introduced to preserve the spectral reflectance characteristics in the input features, ensuring accurate attention-based selection. We also incorporate prior knowledge of vegetation indices into the deep learning framework, using a gating mechanism to select the most relevant biophysical features related to photosynthesis. Additionally, power compression is applied during preprocessing to enhance weak signal features and improve model performance. Our research objectives are as follows: (1) to explore the feasibility and reliability of using deep learning for mining photosynthetic sensitive bands and vegetation indices; (2) to investigate the utility of spectral power compression and verify the stability and applicability of our developed model under different spectral resolutions; (3) to evaluate the performance of our developed model in estimating photosynthetic capacity and illustrate its advantage over the traditional models.

## 2 Materials and methods

### 2.1 Data acquisition and processing

We collected samples of rice and wheat from two distinct experimental locations. The rice samples were grown in Fumin Village, Hengsha Township, Chongming District, Shanghai, China (31.34°N, 121.84°E) from May to November 2022. This area has a subtropical monsoonal climate with an average annual temperature of 15.4°C and annual precipitation of around 1,100 mm. No special irrigation or fertilizer treatments were applied to the experimental field. The wheat samples were grown in Wuhan, Hubei Province, China (30.54°N, 114.36°E) from November 2022 to June 2023. This region has a north subtropical monsoonal climate, with an average annual temperature of 15.8°C to 17.5°C and annual precipitation of 1,150 mm to 1,450 mm. To ensure comprehensive coverage, we collected samples of both rice and wheat throughout the entire growth period. The geographic location of the experimental area is shown in **Supplementary Figure S1**, and the experimental data collection images are displayed in **Supplementary Figure S2**.

#### 2.1.1 Gas exchange measurement and photosynthetic capacity acquisition

The photosynthetic parameters *V_cmax_* and *J_max_* were obtained from leaf gas exchange measurements using a portable gas exchange system, LI-6800 (LI-COR, Lincoln, NE, USA). The system recorded the response of photosynthetic rate (*A*) to a series of intercellular CO_2_ concentrations (*C_i_*). The leaf chamber temperature was adjusted to match the temperature of the leaves. A full-span calibration for CO_2_, water, and gas flow rate and minimal slope search were performed before each curve measurement. The relative humidity inside the leaf chamber was manually set to match the actual humidity. The photosynthetic measurements were taken at a saturation light intensity of 2000 μmol m^-2^ s^-1^ for rice and 1800 μmol m^-2^ s^-1^ for wheat. The light intensity was determined from the preliminary experiments of the assimilation rate - light intense (*A-Q*) response curve. Gas exchange measurements were conducted on fully expanded leaves from the upper, middle, and lower layers of each rice and wheat plant. The dynamic assimilation technique (DAT) (Stinziano et al., 2019) was employed, with an initial CO_2_ concentration of 100 ppm and a final concentration of 1100 ppm. Due to the significant assimilation shifts caused by high CO_2_ change rates, the ramp rate for CO_2_ changes was set at 100 ppm/min (Stinziano et al., 2017 (Stinziano et al., 2019; Saathoff and Welles, 2021). To determine *V_cmax_* and *J_max_*, Farquhar-von Caemmerer-Berry (FvCB) model (Farquhar et al., 1980; Bernacchi et al., 2001) was fitted to the collected *A-C_i_* curves (Sharkey et al., 2007). The most widely accepted use for the *A-C_i_* curve obtained from DAT is to estimate *V_cmax_* and *J*, and those values are closely aligned between the standard and DAT approaches (Stinziano et al., 2017; Taylor and Long, 2019). We did not consider the effect of mesophyll conductance limitation, consistent with previous studies (Drake et al., 2017; Heckmann et al., 2017; Rogers et al., 2017; Kumarathunge et al., 2019; Salmon et al., 2020; Saathoff and Welles, 2021; Deng et al., 2024). The fitting analysis of the *A-C_i_* curves was conducted using the “plantecophys” package (Duursma, 2015) in the R platform (https://bitbucket.org/remkoduursma/plantecophys). All photosynthetic parameters were normalized to 25 °C .

#### 2.1.2 Spectral data acquisition and processing

The spectral data were collected using a Specim-IQ hyperspectral camera (Oulu, Finland, Behmann et al., 2018), which captured hyperspectral images of each detached leaf. The push-broom camera recorded spectral reflectance in a continuous wavelength ranging from 400 nm to 1000 nm with a spectral resolution of 3.5 nm. Two 150W halogen lamps which cover the 400-1000 nm wavelength range were fixed beside the camera as light sources. A tripod supported them to capture images at a distance of 0.5 m from the leaves. Each scan consisted of 512 spatial channels along the rows. A white panel with 99% reflectance (Spectralon, Labsphere Inc., North Dutton, NH, USA) was placed horizontally next to the leaves and scanned along with the plant leaves in the collected hyperspectral images. The exposure time was adjusted to avoid sensor saturation. Image acquisition and storage were completed within three minutes for each image. The acquired images were processed by applying a mask calculation to remove the background. The mask calculation was performed by segmenting the leaf from the surrounding background based on color thresholds. The spatial dimension of hyperspectral images after mask calculation was consistent with the spatial area measured by the LI-6800. The spatial dimensions of the leaves were averaged to obtain spectral reflectance. Then, we used Savitzky-Golay (SG) filter to remove noise (Schafer, 2011). The window length was set to 21, and the polynomial fitting order was 2.

Based on the SG filter, we applied different power compression (POC) ratios to enhance the spectral signals (Li et al., 2021a, b). Power compression is a nonlinear transformation technique commonly used in speech spectral signal processing to adjust the range and distribution of signal values, especially when the original signal exhibits wide variations in magnitude (Yu et al., 2022; Ochieng, 2023; Wen and Verhulst, 2023; Abdulatif et al., 2024). During network training using mean square error (MSE) as a criterion, the optimization process tends to favor areas with higher spectral values. This focus can obscure finer details in regions with lower values, such as the visible light spectrum. By compressing the reflectance values, we anticipated capturing more intricate information in weak areas such as visible bands region. This method can potentially enhance the quality of spectral feature extraction.

We only applied power compression to the reflectance values (amplitude). We did not compress the relative trends (phase). This transformation equalizes the importance of all spectral bands by compressing the highly variable reflectance values, especially in regions with uneven distributions. This compression is particularly beneficial for highlighting weaker signals that may otherwise be overshadowed by stronger reflectance values in certain regions, such as the near-infrared spectrum, which often dominates hyperspectral data. A generalized compression method was employed. The calculation formula is as follows:


(1) $$X^{c}=|X{|}^{\beta }$$

In this study, we considered power compression transformation in the range of 0.1-2.0 proportions. The transformations are denoted by the adjustable compression parameter β∈(0,1). A smaller value of β corresponds to stronger compression. Conversely, when β>1, it represents an inflation transformation that amplifies information in regions of high values. For β=1, no transformation is applied. The compressed spectral information is represented as $X^{c}$ .

The application of power compression impacts model performance by adjusting sensitivity to spectral features. By amplifying weak spectral signals, especially in regions like the visible spectrum, it improves the ability of the model to detect subtle variations. Additionally, power compression helps balance the focus across spectral bands, preventing overfitting to dominant signals and ensuring the model captures important features in weaker bands.

To validate the applicability of the model at different resolutions, spectral resampling was conducted using cubic spline interpolation (McKinley and Levine, 1998). Given data bands (*x_0_*, *R_0_*), (*x_1_*, *R_1_*), …, (*x_n_*, *R_n_*), we interpolated between every two bands. For the interval [*x_i_, x_i+1_*], the form of the reflectance curve *R_i_(x)* is:


(2) $$R_{i}\left(x\right)=a_{i}+b_{i}\left(x-x_{i}\right)+c_{i}{\left(x-x_{i}\right)}^{2}+d_{i}{\left(x-x_{i}\right)}^{3}$$

where *a_i_*, *b_i_*, *c_i_*, *d_i_* are coefficients that need to be determined based on boundary conditions and continuity conditions. The entire reflectance curve is composed of these locally defined cubic polynomials. All of our resampling operations were based on the original data and ensure consistency with the original data. The spectra were resampled to 60, 120, 180, 204, 240, 300, 400, 500 and 600 bands.

We also used spectral data from previous studies as one of our validation datasets, which included experimental data covering 350-2500 nm, with 2151 spectral bands across 583 samples (Meacham-Hensold et al., 2020; Furbank et al., 2021; Kumagai et al., 2022). Data spanning 601 spectral bands from 400 to 1000 nm were also utilized for comparison. Related results can be seen in **Supplementary Figure S6**.

### 2.2 Traditional vegetation index calculation

This study used several traditional vegetation indices to examine their correlation with photosynthetic capacity (**Table 1**). Spectral indices associated with leaf pigment such as chlorophyll content have been frequently employed in phenotypic analysis of plant photosynthesis. For instance, the SIPI, also known as the chlorophyll index (Dash and Curran, 2007), is linked to chlorophyll content. The indices based on the crucial pigment chlorophyll content may serve as reliable indicators of photosynthetic capacity (Croft et al., 2017). In addition, the simple ratio vegetation index (SR) and the modified normalized difference vegetation index (mNDVI) were also used for estimating photosynthetic capacity (Fu et al., 2020).

**Table 1:** Spectral indices utilized in this study.

Vegetation indices	Formula	References
simple ratio (SR)	$R_{\lambda_{1}}/R_{\lambda_{2}}$	(Clevers and Kooistra, 2011)
modified normalized difference index (mNDVI)	$\left(R_{\lambda ref}-R_{\lambda 1}\right)/\left(R_{\lambda ref}+R_{\lambda_{2}}\right)$	(Gitelson and Merzlyak, 1994)
structure insensitive pigment index (SIPI)	$\left(R_{\lambda ref}-R_{\lambda 1}\right)/\left(R_{\lambda ref}-R_{\lambda_{2}}\right)$	(Curran, 1994)

The wavelength *λ_ref_*, denoting 440 nm in the blue spectral range, is based on the study by Jay et al. (2017). *R* represents the reflectance of any band. *λ_1_* and *λ_2_* represent any band within the 400–1000 nm range.

### 2.3 The classical machine learning algorithms

#### 2.3.1 Support vector regression

The SVR methodology first maps the input data to a higher dimensional (possibly infinite) kernel feature space by means of a nonlinear mapping $\phi :{\mathbb{R}}^{N}\to ℋ$ and then solves a linear model there (Camps-Valls et al., 2006):


(3) $${\hat{y}}_{i}=f\left({\text{x}}_{i},w\right)=\phi^{T}\left({\text{x}}_{i}\right)w+b$$

where ${\hat{y}}_{i}$ are the estimations of $y_{i}$ ; **w** is a weight vector in the feature space, and b is the bias term in the regression. The SVR was implemented using the Python library sklearn.

#### 2.3.2 Partial least squares regression

The PLSR model has been applied to estimate leaf photosynthetic capacity (Serbin et al., 2012; Ainsworth et al., 2014). PLSR is a bilinear regression technique that aims to reduce a large set of collinear spectral variables into a smaller set of orthogonal components (Wold et al., 2001). The explanatory variables *V_cmax_* and *J_max_* are projected into a new space. A linear regression model is then fitted between these independent variables and the predicted variables in the new projection space. The computational formula for PLSR is as follows:


(4) $$y=\sum_{i=1}^{n}\gamma_{i}*p_{i},i=1,2,\ldots n.$$

(5) $$p_{i}=\sum_{j=1}^{d}\lambda_{j}*x_{j},i=1,2,\ldots n.$$

The variable *y* denotes *V_cmax_* and *J_max_*. The number of latent variables used for regression is represented by *n*. The regression coefficient is denoted by *γ*. The latent components computed from the original input measurements *x* are denoted by *p. d* is the dimension of the input data. *λ* represents the transformed latent components, which are calculated as *x^T^x*. The PLSR was implemented using the Python library sklearn. The number of principal components was optimized through a grid search of 10 to 15 to find the best value.

#### 2.3.3 One-dimensional convolution

Given the processing of spatially averaged spectral data, a one-dimensional convolutional network has been utilized as one of the baseline models. The OneDCNN often incorporates a hierarchical structure that captures increasingly complex feature patterns. The OneDCNN uses a learned weight filter to slide across the input length at each layer. This sliding filter strategy enables the network to detect patterns found in one part of the sequence and applies these patterns to other parts of the sequence. To further enhance the ability of the network to capture a larger receptive field, we incorporated dilated one-dimensional convolution. The mathematical operation for computing the one-dimensional convolution of input is expressed in the following equation:


(6) $$X_{C_{\circ }}=\phi \left(\sum^{}\left(W\cdot X_{C_{i}}+b\right)\right)$$

where *W* is the parameter matrix of the convolutional kernel; *X_Ci_* is the input feature; *C_i_* represents the input channel; *b* represents the learnable bias; ϕ denotes the activation function; and *C_o_* represents the output channel.

The parameter settings for the OneDCNN model were determined based on previous study (Furbank et al., 2021). The input to the network was a reflectance sequence of size 1×204. The sequence first underwent an initial layer of average pooling, with a kernel size of 10, a stride of 10, and a padding of 2. Two layers of one-dimensional convolution were then applied. The first convolutional layer had an input channel of 1, output channels of 50, a kernel size of 5, and a dilation factor of 1. The second convolutional layer had input channels of 50, output channels of 50, a kernel size of 5, and a dilation factor of 2. Finally, the output passed through two fully connected layers. The number of neurons in the first fully connected layer ranged from 400 to 1000. The number of neurons in the second fully connected layer ranged from 1000 to 1.

#### 2.3.4 Vegetation indices convolution

IndiceCNN, built for dealing with one-dimensional reflectance in our previous research (Deng et al., 2024), extracts the abstract features from reflectance using convolution and pooling operators and then feeds these features into a computation formula of vegetation indices. In this study, the addition-multiplication calculation was incorporated into the IndiceCNN model.


(7) $$R_{i}=\sigma \left(\sum^{}\left(W_{c}\cdot X_{i}+b\right)\right)$$

(8) $$O=\sum^{}\left(W_{l}\cdot \frac{R_{1}+R_{2}}{R_{3}\times R_{4}}\right))$$

*X_i_* denotes the input. *W_c_* represents the weight matrices of different convolutional units. *σ* is the sigmoid function. *W_l_* represents the weight matrices of various linear layers. *R_i_* represents the spectral features after convolution layers. *O* denotes the output.

### 2.4 Design of the Indexfindnet model

**Figure 1:**
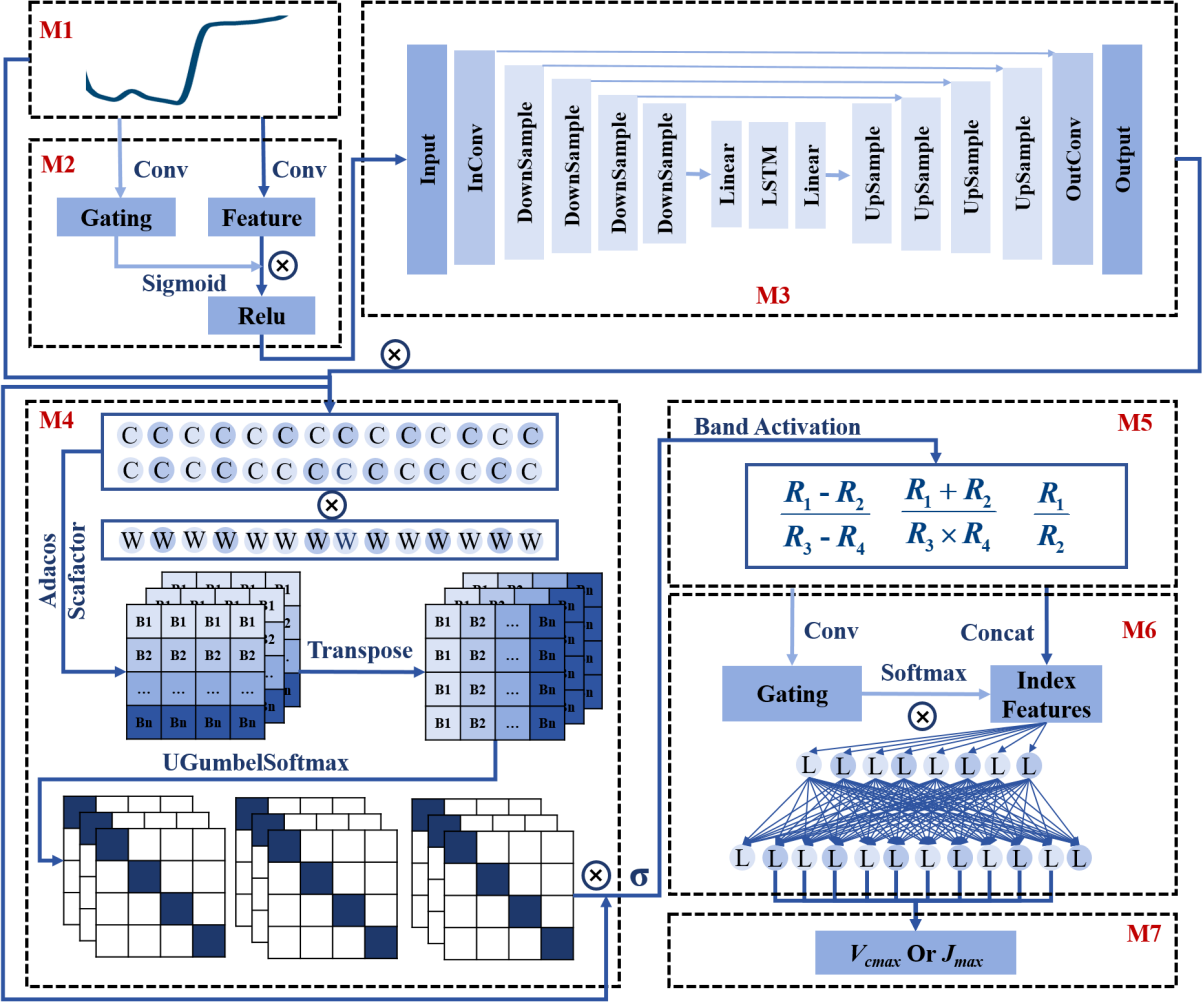
The architecture of the Indexfindnet model. M1 denotes the input reflectance. M2 denotes the gated convolutional module GCONV. M3 denotes the encoder-decoder module UCRN. M4 is the global sensitive band search module NonlocalBandAttention. M5 represents the Vegetation Index calculation module Indexcal. M6 is the module for choosing the important index form features. M7 is the output. C represents the convolutional layer. L represents the linear layer. W denotes the weight matrices of cosine similarity. Adacos Scafactor is the adaptive cosine scaling factor.

To fully leverage spectral dimension information and investigate the spectral sensitive bands and vegetation index with the latent mechanism of photosynthesis, we proposed a novel architecture that employed multiple modules coupled with different branches (**Figure 1**). The subsequent sections would provide detailed descriptions of the network structure, loss function, and solutions to address overfitting issues.

#### 2.4.1 The architecture of Indexfindnet

The framework structure of the Indexfindnet model was shown in **Figure 1**. The first operation of the network structure was the feature extraction and noise removal module “Mask”. This module consisted of two components. The first component was a gated convolution unit named M2-GCONV. Traditional convolution calculated all features as valid values and extracted local features through a sliding window. However, hyperspectral reflectance data contained large amounts of information with high correlations and redundancies between different spectral bands. Therefore, we utilized a technique called gated convolutional units. This method provided a learnable dynamic feature-selection mechanism for each band in every channel (Yu et al., 2019). The calculation formula is as follows:


(9) $$Gating=\sum^{\;}W_{c}•I_{b}$$

(10) $$Feature=\sum^{\;}W_{c}•I_{b}$$

(11) O=ϕ(Feature⊙σ(Gating))

where ϕ refers to the ReLU activation function; σ is the sigmoid function; $I_{b}$ denotes the input features; $W_{c}$ represents different convolution kernels; O represents the output; and ⊙ denotes the matrix multiplication.

In the second part, M3-UCRN combined multi-level feature extraction with an encoding-bottleneck-decoding process (Ronneberger et al., 2015). This architecture effectively captures crucial features in the spectral dimension by learning a compact representation of the data that filters out noise. By compressing the input into a lower-dimensional space, it suppresses irrelevant or noisy components, while preserving the original data structure and avoiding distortions or compression of spectral bands (Chiang et al., 2019; Casas et al., 2021; Konstantinova et al., 2021). The lower-level features were directly connected to the higher-level features to preserve and restore fine-grained details. By gradually reducing the feature dimension, high-level semantic features were extracted through the downsampling operation. After the features were compressed by a linear layer, they were fed into a recurrent neural network (LSTM) for sequence feature learning (Sherstinsky, 2020). The output of the LSTM was further expanded through a linear layer. The data size was gradually restored via upsampling operations during the decoding process. This module ultimately achieved fine-grained feature extraction. The core formulas of the UCRN module are as follows:


(12) $$O_{Maxpool}=max\left(I_{b},I_{b+1}\right)$$

(13) $$O_{Encoder}=\phi \left(\sum^{\;}(W_{c}•O_{\text{Max}pool}\right))$$

(14) $$I_{b}=\sum^{\;}(W_{c}•\sum^{\;}\left(W_{l}•O_{Encoder}\right))$$

(15) $$O_{b-1}=\sigma \left(\sum^{\;}\left(W_{c}•I_{b-1}\right)\right)$$

(16) $$O_{b}=\sigma \left(\sum^{\;}\left(W_{c}•I_{b}\right)\right)$$

(17) $$O_{bottleneck}=\sigma \left(\sum^{\;}(W_{c}•\left(O_{b}+O_{b-1}\right)\right))$$

(18) $$I_{decoder}=\sum^{\;}(W_{c}•\sum^{\;}\left(\left(W_{l}•O_{bottleneck}\right)\right))$$

(19) $$O_{Decoder}=\phi \left(\sum^{\;}\left(W_{c}•\sum^{\;}\left(W_{c}^{T}•I_{Encoder}+O_{Encoder}\right)\right)\right)$$

where the input is denoted as *I_b_*; *W_c_* represents the weight matrices of different convolutional units; *W_l_* represents the weight matrices of various linear layers; *W_T_* denotes the transposition of a weight matrix. The activation function is ϕ , specifically the Rectified Linear Unit (ReLU) activation function. The sigmoid function is denoted as *σ*. Additionally, *I_decoder_*, *O_encoder_*, and *O_decoder_* correspond to the input of the decoding layer, the output of the encoding layer, and the output of the decoding layer, respectively.

The calculation formula for the Mask module of the input data is as follows:


(20) O=I×Mask(I)

where *I* denotes to the input data; Mask refers to the feature extraction module; and *O* refers to the output.

Then, we moved on to the most critical module, the Indexfind module. The core idea of this module was to leverage the network to automatically identify sensitive bands and vegetation indices from the spectral data. It consisted of two layers. The first layer aimed to identify sensitive bands from the full spectral sequence by conducting a comprehensive search across all spectral bands using the M4-NonlocalBandAttention module. This module utilizes a global attention mechanism and one-hot encoding vectors corresponding to each spectral band, allowing precise identification of the most relevant bands. Several improvements were introduced in the M4-NonlocalBandAttention module to enhance its performance over traditional attention mechanisms. Firstly, two layers of dilated convolutions were added before the global operation of attention to enhance feature extraction and eliminate spatial redundancy in spectra. Secondly, instead of a standard linear transformation, we used a global cosine similarity calculation to better capture the relationships between spectral bands across the entire wavelength range. This technique involves normalizing both the input features and the cosine similarity weight matrices, and then multiplying them. This approach helps better capture the relationships between spectral bands and enables more accurate alignment across the entire wavelength range. Thirdly, the similarity matrix of the attention weight matrix was replaced with a single sequence activation vector as a global alignment of weights. Fourthly, to further enhance band separability, particularly when dealing with noisy or overlapping spectral data, we introduced an adaptive cosine scaling factor. This factor multiplies with the band activation vectors, refining the selection of relevant bands by improving the separability and reducing noise overlap, which is crucial for accurate feature extraction (Zhang et al., 2019; Wilkinghoff, 2021). Fifthly, the softmax function was replaced with the modified uniform gumbel softmax function, which determined a specific band rather than the probability of a band. We replaced the gumbel noise with uniform noise, which had a certain regularization effect. The gumbel softmax function also enabled gradient-based updates during backpropagation by making the discrete distribution sampling process differentiable (Jang et al., 2017). Gumbel softmax introduced randomness. We employed the second step to control the range of input data. At the same time, we introduced a shrinking factor in gumbel softmax to reduce the influence of noise. The model results were stable. Finally, the feature output was passed through a sigmoid function and normalized to a range of 0-1 for physical compatibility with reflectance. The second layer of the Indexfind module used the sensitive bands identified by the M4-NonlocalBandAttention module to perform vegetation index calculations. These sensitive bands, selected through the attention mechanism, were expected to capture key spectral features that are most relevant for photosynthetic capacity estimation. The identified bands were then used to calculate various vegetation indices, which were designed to capture the non-linear relationships between spectral bands that reflect photosynthetic activity. We have constructed vegetation index formulas to calculate the non-linear combination of addition, subtraction, multiplication, and division. These formulas served as the main framework of the M5-IndexCal module (Equations 27–29).


(21) $$Q=\phi \left(\sum^{\;}\left(W_{c}•I_{b}\right)\right)$$

(22) $$V=\phi \left(\sum^{\;}\left(W_{c}•Q\right)\right)$$

(23) $$K=\alpha \sum^{\;}\left(\frac{W_{l}}{\left|W_{l}\right|}•\frac{V}{\left|V\right|}\right)$$

(24) $$A=UGumbelSoftmax\left(K^{T}•{\sqrt{D}}^{-1}\right)$$

(25) $$p_{i}^{'}=\frac{\text{exp}\left((g_{i}+\log\pi_{i})/\tau \right)}{\sum_{j}\text{exp}\left((g_{i}+\log\pi_{i})/\tau \right)}$$

(26) $$O_{a}=\sigma \left(A•I_{ref}\right)$$

(27) $$Index_{1}=\frac{R_{1}-R_{2}}{R_{3}-R_{4}}$$

(28) $$Index_{2}=\frac{R_{1}+R_{2}}{R_{3}\times R_{4}}$$

(29) $$Index_{3}=\frac{R_{1}}{R_{2}}$$

*I_b_* denotes the input of M4. *I_ref_* denotes the reflectance data. *W_c_* represents the weight matrices of different convolutional units. *W_l_* represents the weight matrices of cosine similarity. α is the adaptive cosine scaling factor. The calculation of the adaptive cosine scaling factor can be found in Equations 35-38. *UGumbelSoftmax* denotes the uniform gumbel softmax. $\pi_{i}$ denotes the input of uniform gumbel softmax. *g_i_* denotes the uniform noise. τ is the temperature factor. $p_{i}^{'}$ denotes the distribution of uniform gumbel softmax. The activation function is denoted by ϕ . *D* represents the data feature dimension. The sigmoid function is denoted as *σ*. The bands $R_{1}$ , $R_{2}$ , $R_{3,}$ and $R_{4}$ are identified by the NonlocalBandAttention module.

Next, the data from these three types of vegetation index features were concatenated. A convolutional layer was employed to extract features. The softmax function was used for index importance scoring. Subsequently, two linear layers were used for photosynthetic capacity estimation. The computation formula can be represented as follows:


(30) $$S=Softmax\left(\sum^{\;}\left(W_{c}•I_{b}\right)\right)$$

(31) $$O=\sum^{\;}(W_{l}•\sum^{\;}\left(W_{l}•\sum^{\;}\left(S•I_{b}\right)\right))$$

where *I_b_* represents the input features; *W_c_* denotes the weight matrix of the linear layer; Softmax denotes the softmax function; *S* denotes the gated ratio; *W_l_* denotes the weight matrix of the linear layer; and *O* represents the predicted photosynthetic capacity.

The integration of attention mechanisms and gumbel softmax enabled the model to effectively prioritize the most relevant spectral bands. The M4-NonlocalBandAttention module captured long-range dependencies to identify sensitive bands, while the gumbel softmax technique facilitated the discrete selection of these bands in a differentiable manner, thereby enhancing model stability and regularization. Furthermore, the incorporation of vegetation indices enabled the non-linear combination of spectral bands, which improved the ability of the model to identify key features related to photosynthetic capacity. The gating mechanism dynamically selected the most informative indices, ensuring the extraction of critical spectral features for accurate estimation. Through these strategies, along with noise reduction and dimensionality reduction, the unified deep learning framework effectively identified meaningful patterns in hyperspectral data, thereby ensuring robust performance and improved generalization across diverse datasets.

#### 2.4.2 Loss function design

The loss function of the model consisted of two components. One component was used for constraining the regression of photosynthetic capacity, which was calculated by MSE, as shown in the following equation:


(32) $$MSE=\frac{1}{n}\sum_{i}^{n}{\left(y_{i}-{\hat{y}}_{i}\right)}^{2}$$

where $y_{i}$ represents the actual value; ${\hat{y}}_{i}$ is the corresponding predicted value; and *n* is the number of samples.

The second component of the loss function was primarily intended for the Mask module. The Mask module functioned as a feature extraction module and should not change the data patterns. We proposed a correlation loss named MaskLoss for the input and output of the Mask module. A smaller MaskLoss value indicated a higher similarity between the input and output features. The MaskLoss was calculated using the following formula:


(33) $$MaskLoss=-\sum_{1}^{n}\left(\frac{\sum_{1}^{n}I\times O}{\sum_{1}^{n}\left|I\right|\sum_{1}^{n}\left|O\right|}\right)/n$$

where *I* represents the input data; and *O* represents the output data after applying Mask.

The total loss was the sum of the two components:


(34) AllLoss=MSE+α∗MaskLoss

where *α* represents the scaling factor for MaskLoss, which was set to 0.05 during model training; and AllLoss refers to the overall model loss.

#### 2.4.3 Solutions to prevent overfitting

Overfitting significantly hindered the ability of the model to generalize effectively to the testing set. To address this issue, we employed three techniques: early stopping, *L_2_* regularization, and dropout.

Early stopping aimed to prevent overfitting by stopping the model training in the early stages (Caruana et al., 2000). This approach prevented the model from continuously learning the noise in the input data. It encouraged the model to focus on mapping higher-level features of the input data and generalize better to the testing dataset. In our case, we trained the model for 1000 epochs. We evaluated the validation dataset every 50 epochs and stopped training when the model did not improve after 500 epochs. We saved the model with the highest validation score throughout the training process. And the results usually occurred before the end of training.

*L_2_* regularization was used to address overfitting (Byrd and Lipton, 2019). We set the initial regularization parameter weight decay to 10^-3^. By incorporating the *L_2_* norm as the regularization term, we obtained an optimized solution with small and proximate, yet non-zero values for each parameter *w* that is associated with the feature. This regularization term also helped prevent the model from becoming complex to fit the training dataset and enhance the generalization capability of the model.

We also incorporated a dropout layer of 20% before the final linear layer to assist the model in avoiding overfitting. Dropout randomly deactivated a percentage of neurons during training. It prevented complex dependencies between neurons from forming. It encouraged neurons to work more independently. This led to simpler mappings from input to output.

Additionally, we added batch normalization layers within the network structure. Batch normalization sped up network convergence as a normalization technique. It also provided some regularization effects (Ioffe and Szegedy, 2015).

#### 2.4.4 Parameter setting and model training

The input size is a 1×204 vector. Firstly, it underwent a gated convolutional unit M2, with an input channel size of 1, an output channel size of 128, a kernel size of 5, a stride of 1, and a padding value of 2. The gated factor is the result of convolution followed by sigmoid. The output shape of the gated convolutional unit is 64×204.

The shape of the input data to the encoding layer is 64×204. Firstly, it was passed through a mapping layer, which consisted of a two-layer one-dimensional convolution. The convolution layer has an input channel size of 64, a convolution kernel size of 5, a dilation factor of 2, a stride of 1, and a padding of 4. Then, the data entered the encoding layer with a pooling kernel size of 2. During the downsampling process, the channel size was doubled. Four downsampling modules were in the encoding layer with a channel size change of [64, 64, 64, 64]. The upsampling, or decoding layer, had the same channel size changes as the downsampling. The upsampling had a scaling factor 2 and utilized the nearest neighbor sampling method. The output of the upsampling was added to the corresponding output of the last encoding layer to prevent gradient disappearance. Finally, it passed through another layer with an input channel size of 64, an output channel size of 1, and a convolution kernel of 1. As a result, the output data shape is 1×204.

The data input shape for the Indexfind module was 1×204. It was simultaneously fed into three branches for index mining. The number of spectral sensitive bands required for the IndexCal module aligned with the number of core search modules in the NonlocalBandAttention module. Within the NonlocalBandAttention module, the attention weight matrix had an input channel of 1, an output channel of 64, a kernel size of 5, a dilation factor of 2, and a padding of 5. The output shape of the global operation was 64×204. Adacos scale factor was calculated by the formulas as follows:


(35) $$\text{S}=\sqrt{2}*\text{ln}\left(bandnum\right)$$

(36) B=S∗x−max(S∗x)

(37) $$B_{avg}=\frac{1}{N}\sum_{i=1}^{N}B_{i}$$

(38) $$\text{α}=\left(\max\left(S*x\right)+\text{ln}\left(B_{Avg}\right)/\text{cos}\left(\frac{\pi }{4}\right)\right)$$

where bandnum denotes the number of bands; *x* denotes the input data; B_avg_ is the normalization of conditional probability; α is the adaptive cosine scaling factor.

After transposing, the uniform gumbel softmax function was applied to obtain globally aligned weights. The shrinking factor of uniform gumbel softmax noise is 10. The global aligned weights were one-hot vectors. These weights were then scaled and multiplied with the input reflectance data before passing through a sigmoid function. The output shape was 1×64. The output of the NonlocalBandAttention module represented the activated reflectance data in sensitive bands. Subsequently, this output was fed into the IndexCal framework, which obtained three vegetation index features. Each vegetation index branch produced an output shape of 1×64.

Next, the data from these three types of vegetation index features were concatenated with a shape of 3×64. Then, two convolutional layers were employed with an input channel of 128, an output channel of 64, and a kernel size of 1. The softmax function was used for index importance scoring. The weighted sum of these index features was computed with a shape of 1×64. Subsequently, two linear layers were used. The first linear layer has an input channel of 64 and an output channel of 128. This dimensionality transformation allowed the output of the hidden linear layer to be mapped to a higher-dimensional space. It can introduce non-linear transformations and enhance the model expressive power. The second linear layer had an input channel of 128 and an output channel of 1. It reduced the high-dimensional features to a lower-dimensional space and extracted the most important and representative features. This combination of dimensionality transformations enabled the model to capture complex features flexibly and efficiently and improved its performance and generalization ability.

The data was randomly split into training, validation, and testing sets, with each set accounting for 80%, 10%, and 10%, respectively. We used the validation and testing sets to evaluate the model. The model was trained on the training set using the RAdam algorithm.

We randomized and divided the training set into mini-batches for network input. The batch size was set to 8 with an initial learning rate 0.001. We implemented the CyclicLR decay strategy. Training stopped upon reaching the maximum number of iterations. The parameter settings of the model can be found in the appendix.

We fed partitioned testing sets into the trained network for forward propagation during validation. Each spectral reflectance was linked to a predicted photosynthetic parameter value. We applied separate models for each of the two photosynthetic parameters. Test results from all deep learning models were averaged over three runs. The deep learning models were built and tested using the PyTorch deep learning framework. We utilized an NVIDIA GeForce RTX 2060 SUPER GPU with 8GB of memory.

### 2.5 Evaluation metrics

The performance of different models was evaluated based on the coefficient of determination (R^2^), root mean square error (RMSE), and mean absolute percentage error (MAPE). A model performed better if it had a higher R^2^ and lower RMSE and MAPE values.


(39) $$R^{2}=1-\sum_{i=1}^{n}{\left(y_{i}-{\hat{y}}_{i}\right)}^{2}/\sum_{i=1}^{n}{\left(y_{i}-y_{m}\right)}^{2}$$

(40) $$RMSE=\sqrt{\sum_{i=1}^{\text{n}}{\left(y_{i}-{\hat{y}}_{i}\right)}^{2}/n}$$

(41) $$MAPE=\frac{100}{n}\sum_{i=1}^{n}\left|\frac{{\hat{y}}_{i}-y_{i}}{y_{i}}\right|$$

where ${\hat{y}}_{i}$ represents the predicted values of Vcmax and Jmax; $y_{i}$ represents the values of Vcmax and Jmax fitted by the A-Ci curve; $y_{m}$ represents the average measured values of Vcmax and Jmax, and n represents the number of samples in the testing set.

## 3 Results

### 3.1 Characteristics of photosynthetic capacity and hyperspectra

**Figure 2:**
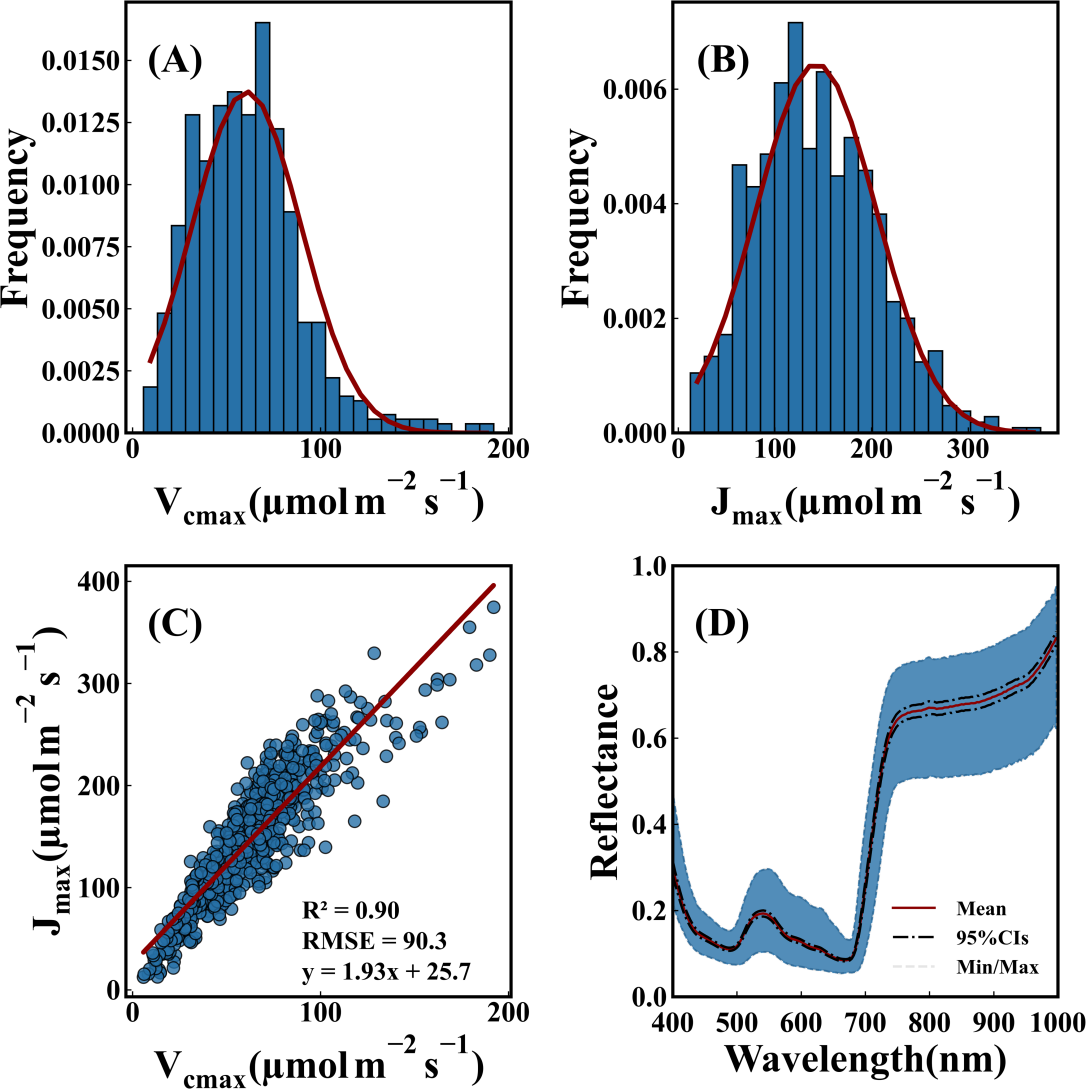
Statistical description of photosynthetic capacity and spectra. **(A)** Distribution of *V_cmax_*. **(B)** Distribution of *J_max_*. **(C)** Correlation between the two photosynthetic parameters. **(D)** Hyperspectral reflectance data of leaf samples. A solid red line represented average reflectance. A black dashed line indicated the 95% confidence interval. Gray dotted lines marked maximum and minimum reflectance values of multi leaf samples.

**Figure 2** illustrates the characteristics of photosynthetic capacity and spectra. *V_cmax_* ranged from 5 to 195 μmol m^-2^ s^-1^ throughout the growth period. *J_max_* ranged from 5 to 350 μmol m^-2^ s^-1^. *V_cmax_* and *J_max_* values were predominantly distributed within the range of 60-100 and 100-200 μmol m^-2^ s^-1^, respectively. The ratio of *J_max_*/*V_cmax_* was 1.93, with a standard deviation of 25.7. These findings aligned with previous studies (Wullschleger, 1993). Furthermore, a strong correlation was observed between *V_cmax_* and *J_max_* (Qian et al., 2021). **Figure 2D** presents the hyperspectral reflectance data of leaf samples from both rice and wheat. Absorption peaks occurred at 410–450 nm and 660–690 nm. A reflection peak appeared at 500–550 nm.

### 3.2 Correlation between traditional vegetation indices and photosynthetic capacity

**Figure 3:**
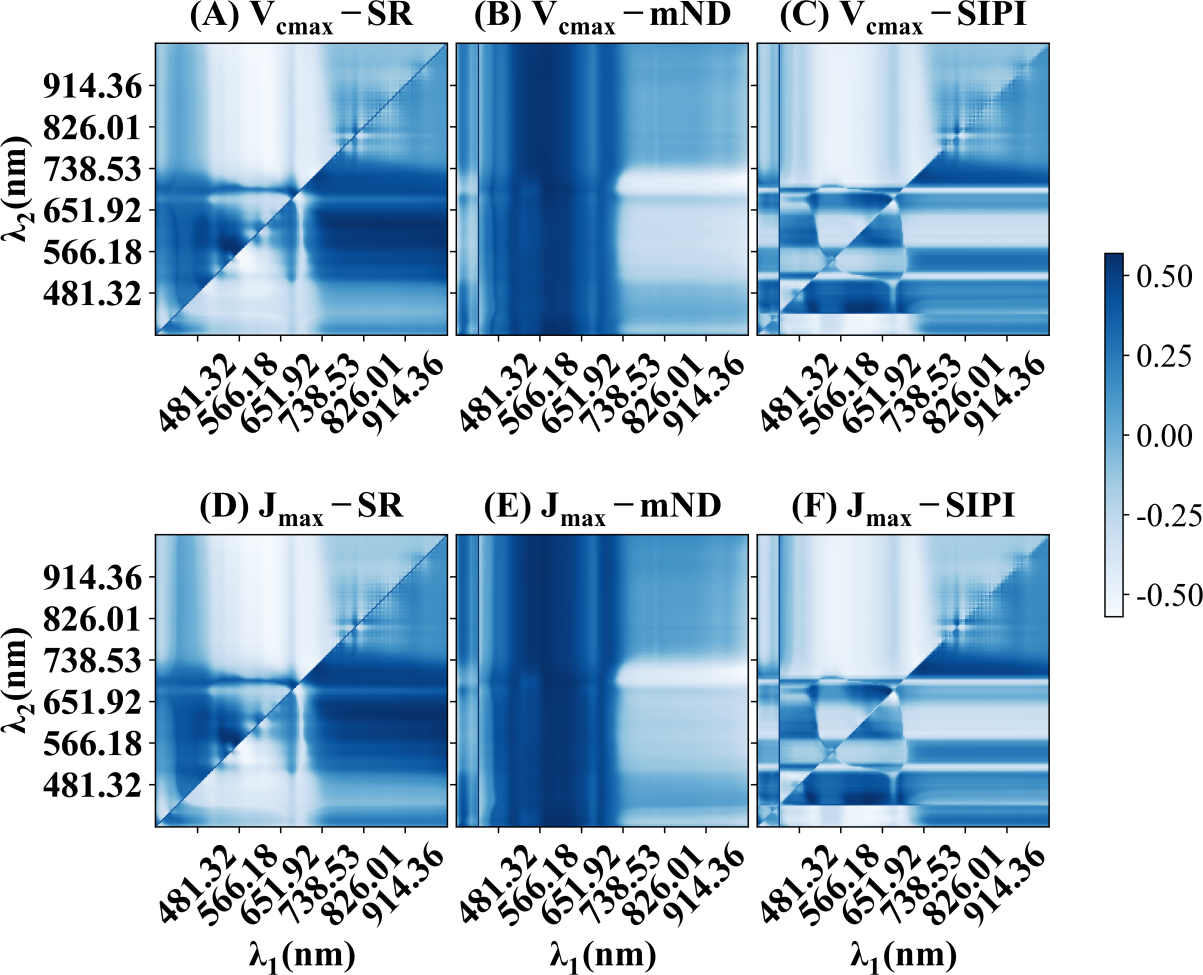
The correlation coefficients (ρ) between *V_cmax_*, *J_max,_* and spectral indices in wheat and rice leaves. **(A, D)** presents simple ratios (SR). **(B, E)** presents modified normalized difference index (mNDVI) **(C, F)** present structure-insensitive pigment index. The equations for these spectral indices can be found in **Table 1**.

**Figure 3** illustrates the correlation coefficients (ρ) between *V_cmax_* and *J_max_* with various traditional spectral indices. High correlation coefficients were observed in several reflectance combinations, indicating potential relationships between the spectral indices and photosynthetic parameters. Specifically, **Figures 3A, B, D, E** highlight spectral regions between 490 to 530 nm and 560 to 660 nm, which are associated with the light absorption properties of chlorophyll and nitrogen content (Carter, 1994; Blackburn, 1998). In contrast, **Figures 3C**, **F** demonstrate that SIPI yielded a correlation coefficient (ρ) below -0.5, with *λ_1_* between 600 and 690 nm and *λ_2_* between 420 and 460 nm. This spectral range corresponds closely to the absorption spectra of total chlorophyll and the absorption properties of PSII and PSI (Laisk et al., 2014), which explained the observed hotspots in **Figures 3C**, **F**. SIPI (**Figures 3C, F**) exhibited the weakest correlation with photosynthetic capacity compared to other spectral indices. It was worth noting that there was a strong correlation between the combination of near-infrared and visible light and photosynthetic parameters, regardless of the spectral index used. Overall, the spectral indices demonstrated moderate correlations with photosynthetic capacity, with the highest correlation coefficient reaching approximately 0.5.

### 3.3 Performance of the Indexfindnet under different power compression spectra

**Figure 4:**
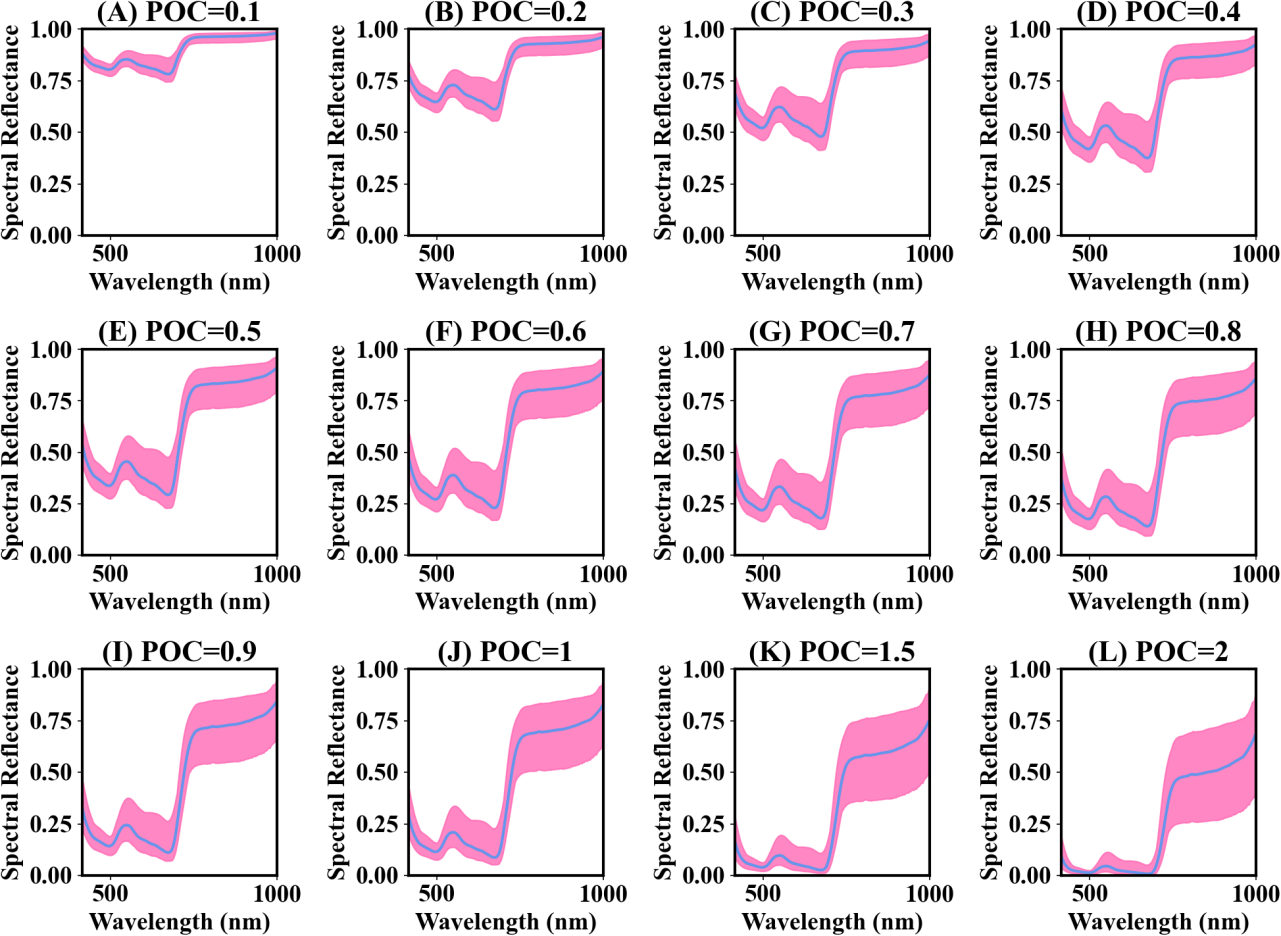
Average power compression (POC) spectra for rice and wheat leaves. ‘POC=0.1’ denotes that the power compression ratio is 0.1. The pink-shaded region represents the difference between the maximum and minimum spectral values. The blue line represents the mean spectral reflectance. **(A–L)** represent different power compression ratio spectra.

**Figure 4** presents the spectra of different power compression ratios. The compressed spectral curves still retained the original trends. The decrease in the difference between the maximum and minimum values within the wavelength range of 400-720 nm was insignificant compared to 720-1000 nm when the compression ratio was less than 1. There was a significant increase in the difference between the maximum and minimum values within the wavelength range of 720-1000 nm with a compression ratio greater than 1 (**Figures 4D–G**). The spectral signals in the visible region were enhanced.

**Figure 5:**
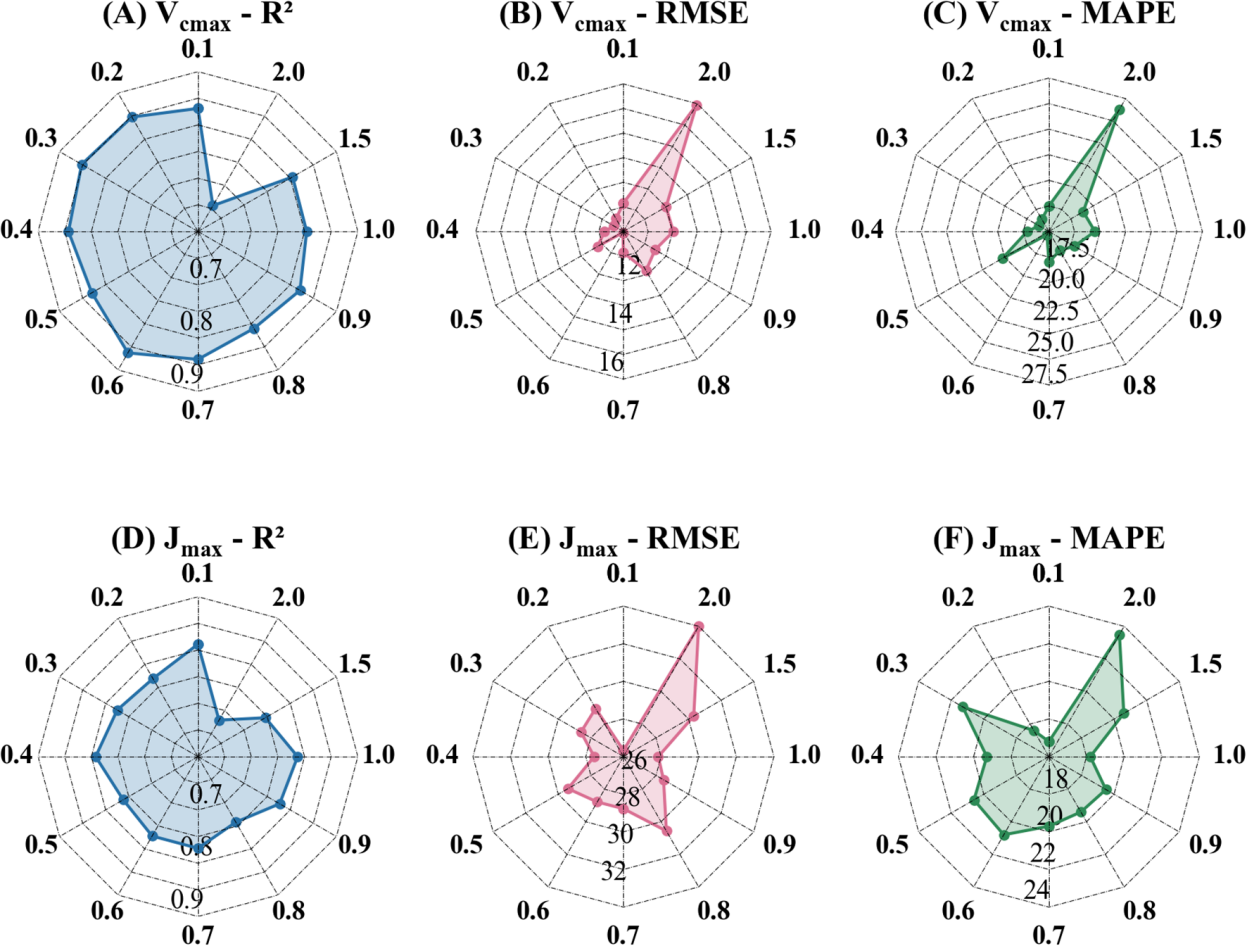
Performance of Indexfindnet in estimating photosynthetic parameters under different compressed spectra. The number around the outer circle represents the power compression ratio. The numbers inside the circle represent the axis labels of each evaluation metric. The unit of RMSE is μmol m^-2^ s^-1^. The value of MAPE represents a percentage. **(A, D)** are the R² values for estimating photosynthetic parameters under different power compression ratios; **(B, E)** are the RMSE values for estimating photosynthetic parameters under different power compression ratios; **(C, F)** are the MAPE values for estimating photosynthetic parameters under different power compression ratios.

This study evaluated the estimation accuracy of the Indexfindnet model using power compression spectra (**Figure 5**). R^2^, RMSE, and MAPE were calculated to assess the accuracy of the model on the validation dataset (**Supplementary Table S1**). The baseline model (Indexfindnet with no power compression, POC ratio=1.0) achieved an R² of 0.82, RMSE of 11.43 μmol m^-2^ s^-1^, and MAPE of 18.9% for *V_cmax_*. For *J_max_*, the model yielded an R² of 0.80, RMSE of 25.75 μmol m^-2^ s^-1^, and MAPE of 16.5%. For the photosynthetic parameter *V_cmax_*, the model based on POC-0.6 (POC ratio=0.6) achieved best performance, with an R^2^ of 0.86, RMSE of 10.10 μmol m^-2^ s^-1^, and MAPE of 15%. The best performance was observed with POC-0.1 (POC ratio=0.1) for *J_max_*, with an R^2^ of 0.81, RMSE of 25.33 μmol m^-2^ s^-1^, and MAPE of 16.8%. POC-2.0 (POC ratio=2.0) had the poorest performance for both photosynthetic parameters. For *V_cmax_*, the R² dropped to 0.66, with an RMSE of 15.94 μmol m^-2^ s^-1^ and a MAPE of 26.0%. For *J_max_*, the performance was similarly lower, with an R² of 0.68, RMSE of 32.99 μmol m^-2^ s^-1^, and MAPE of 23.5%. The Indexfindnet model with POC ratios less than 1 consistently outperformed the baseline model, which was based on the uncompressed spectra. The R² values for the compressed models ranged from 0.83 to 0.86, with RMSE values between 10.05 μmol m^-2^ s^-1^ and 11.22 μmol m^-2^ s^-1^, while the baseline model achieved an R² of 0.82 and an RMSE of 11.43 μmol m^-2^ s^-1^ for *V_cmax_*.

### 3.4 Performance of Indexfindnet and traditional methods for leaf photosynthetic capacity estimation

**Table 2** presents the performance comparison of the classical machine learning model and the Indexfindnet. The Indexfindnet model had a modest parameter count, remaining under one million. We can observe that the deep learning method performed better than traditional machine learning methods. The R^2^ values were 0.20-0.39 higher than those of the PLSR model. Specifically, the R² values for *V_cmax_* and *J_max_* in the Indexfindnet model were 0.86 and 0.81, respectively, significantly higher than the PLSR model, which achieved 0.58 for *V_cmax_* and 0.56 for *J_max_*. The RMSE and MAPE values were lower than the PLSR model by 6.1-12.48 μmol m^-2^ s^-1^ and 9%-15%, respectively. Before feeding the spectral data into the Indexfindnet model, POC was used to reduce the reflectance difference between visible light and near-infrared regions. This preprocessing step allowed the model to better focus on the local details of visible light. The compressed spectral data performed better across different models. Indexfindnet demonstrated the most impressive results in power compression spectra among the models, with the R² values for *V_cmax_* and *J_max_* reaching 0.86 and 0.81, respectively, and an average absolute error of approximately 15% for *V_cmax_* and 17% for *J_max_*.

**Table 2:** The performance of each model in estimating the photosynthetic capacity of maximum carboxylation rate and maximum electron transfer rate.

Model	Process	Params	*V_cmax_*				*J_max_*			
		(M)	R^2^	RMSE	Preds	MAPE	R^2^	RMSE	Preds	MAPE
SVR	SG		0.43 ± 0.03	20.59 ± 0.59	60.56 ± 26	0.28 ± 0.01	0.53 ± 0.01	39.99 ± 0.22	142.50 ± 54	0.27 ± 0.01
SVR	SG-POC		0.42 ± 0.02	20.68 ± 0.44	61.01 ± 25	0.27 ± 0.01	0.56 ± 0.02	38.68 ± 0.18	142.22 ± 48	0.26 ± 0.02
PLSR	SG		0.47 ± 0.02	19.71 ± 0.35	58.62 ± 25	0.30 ± 0.01	0.58 ± 0.04	37.81 ± 1.89	141.58 ± 53	0.27 ± 0.02
PLSR	SG-POC		0.50 ± 0.01	19.23 ± 0.32	58.06 ± 26	0.29 ± 0.02	0.61 ± 0.01	36.96 ± 0.44	140.69 ± 50	0.26 ± 0.01
OneDCNN	SG	0.42	0.75 ± 0.02	13.61 ± 0.41	57.87 ± 23	0.21 ± 0.01	0.78 ± 0.01	27.46 ± 0.36	140.60 ± 49	0.19 ± 0.01
OneDCNN	SG-POC	0.42	0.79 ± 0.02	12.11 ± 0.37	57.52 ± 25	0.20 ± 0.00	0.78 ± 0.01	27.36 ± 0.32	140.54 ± 49	0.18 ± 0.01
IndiceCNN	SG	0.28	0.83 ± 0.02	11.06 ± 1.16	56.62 ± 22	0.18 ± 0.02	0.80 ± 0.01	25.41 ± 0.19	139.39 ± 49	**0.16** ± 0.01
IndiceCNN	SG-POC	0.28	0.84 ± 0.01	11.01 ± 0.25	56.44 ± 22	0.17 ± 0.01	0.80 ± 0.01	25.39 ± 0.98	139.01 ± 49	**0.16** ± 0.01
Indexfindnet	SG	0.63	0.82 ± 0.01	11.42 ± 0.37	56.70 ± 22	0.19 ± 0.01	0.79 ± 0.01	26.85 ± 0.16	136.17 ± 49	0.18 ± 0.01
Indexfindnet	SG-POC	0.63	**0.86** ± 0.01	**10.05** ± 0.42	57.37 ± 22	**0.15** ± 0.02	**0.81** ± 0.01	**25.33** ± 0.34	137.06 ± 49	0.17 ± 0.01

The unit of RMSE is μmol m^-2^ s^-1^. The value of MAPE represents a percentage. For example, a MAPE with a value of 0.30 represents 30%. Bolding indicates the best performance. SG represents the Savitzky-Golay filtering. POC represents power compression. “Params” refers to the number of model parameters, and “M” indicates that the number is expressed in millions. The value before ± is the mean, and the value after ± is the standard deviation. The results were averaged through three runs across randomly split validation set. Preds represents the values of *V_cmax_* and *J_max_* predicted by the model.

### 3.5 Photosynthesis-sensitive bands and vegetation indices discovered by Indexfindnet

**Figure 6:**
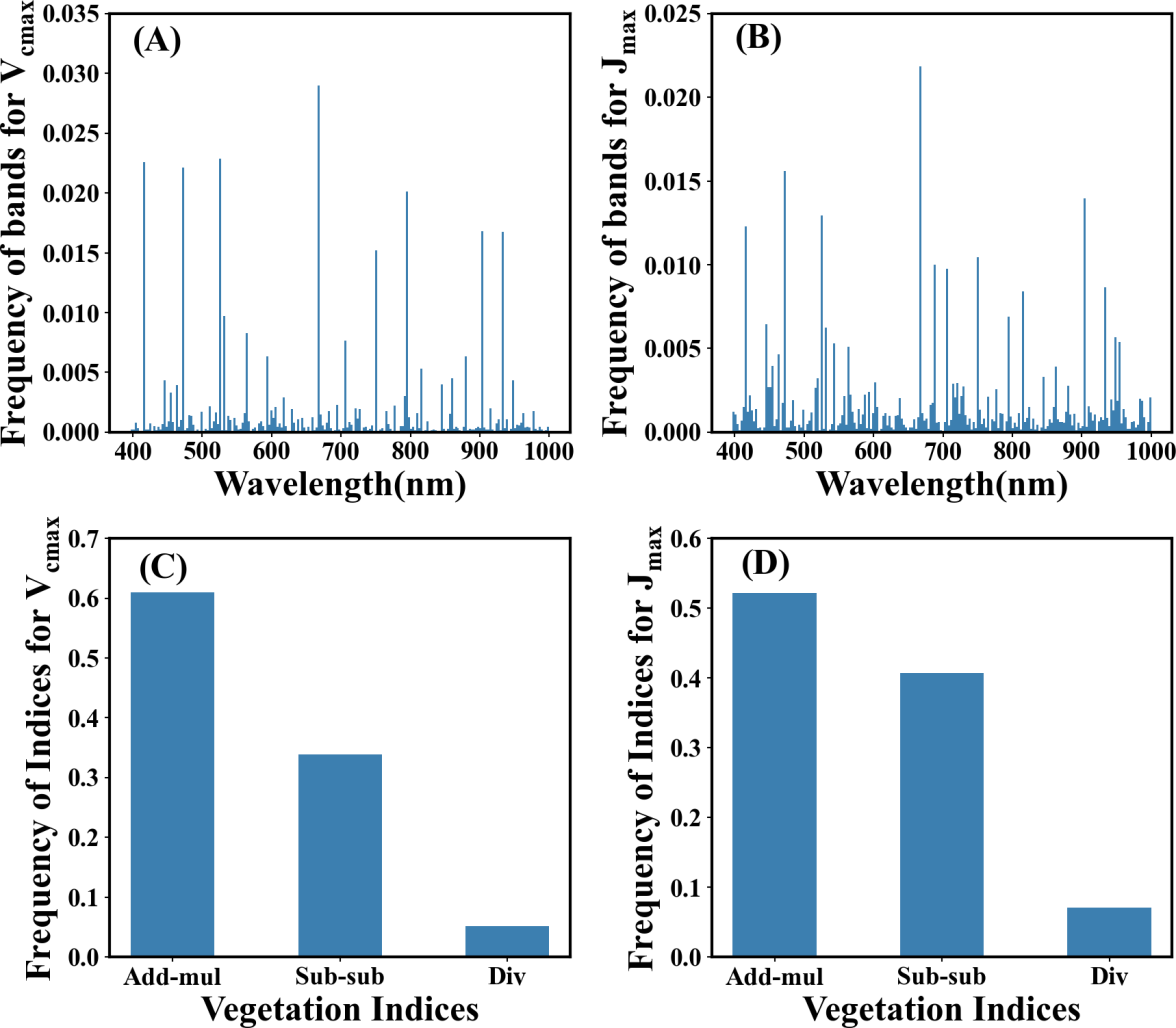
The distribution of the sensitive bands and index formulas for photosynthetic capacity *V_cmax_* and *J_max_* identified by the Indexfindnet model. The vertical axis represents the frequency from all channels at which the wavelength or indices were identified by the best model. **(A, B)** represent the distribution of sensitive bands, and **(C, D)** represent the frequency of vegetation indices.

Sensitive bands significantly contributed to the prediction of photosynthetic capacity. They can be identified through weight analysis of the NonlocalBandAttention module from the best trained model which has the highest accuracy. The results of the search for characteristic wavelengths for photosynthetic capacity are shown in **Figures 6A, B**. The wavelengths at 410-470 nm, 510-530 nm, and 660-690 nm play a crucial role in predicting the photosynthetic capacity. Other wavelengths have little impact on the photosynthetic capacity. The spectral characteristic wavelengths sensitive to photosynthetic capacity were ranked based on search numbers. The top eight wavelengths for *V_cmax_* were 667, 525, 415, 471, 795, 905, 935 and 750 nm. The top eight wavelengths for *J_max_* were 667, 471, 905, 525, 415, 750, 688, and 706 nm.

The ranking of the spectral indices discovered through automatic model search can be seen in **Figures 6C, D** and **Table 3**. The most effective vegetation index formula identified for both *V_cmax_* and *J_max_* was $\frac{R_{nir}+R_{g/b}}{R_{nir}\times R_{r}}$ (**Table 3**). The computation between the near-infrared wavelength and the shorter wavelength is important regardless of the type of index (**Table 3**).

**Table 3:** Spectral indices searched out by the Indexfindnet.

Trait	Index1	Index2	Index3	Index4	Index5	Index
*V_cmax_*	$\frac{R_{795}+R_{525}}{R_{905}\times R_{667}}$	$\frac{R_{471}+R_{525}}{R_{905}\times R_{667}}$	$\frac{R_{795}+R_{525}}{R_{935}\times R_{667}}$	$\frac{R_{795}+R_{415}}{R_{935}\times R_{667}}$	$\frac{R_{845}-R_{616}}{R_{560}-R_{705}}$	$\frac{{\text{R}}_{nir}+R_{g/b}}{R_{nir}\times R_{r}}$
*J_max_*	$\frac{R_{795}+R_{415}}{R_{543}\times R_{667}}$	$\frac{R_{471}+R_{525}}{R_{905}\times R_{750}}$	$\frac{R_{471}+R_{525}}{R_{905}\times R_{667}}$	$\frac{R_{795}+R_{525}}{R_{935}\times R_{750}}$	$\frac{R_{815}-R_{420}}{R_{593}-R_{726}}$	$\frac{R_{nir}+R_{g/b}}{R_{nir}\times R_{r}}$

Index1 refers to the most frequently searched index by the model, Index2, Index3, Index4, and so on in a similar manner. Index refers to the universally summarized indices derived. *R_nir_* represents the reflectance of near-infrared bands. *R_g/b_* represents the reflectance of green or blue bands. *R_r_* represents the reflectance of red bands.

Further validation was conducted to investigate the effectiveness of the identified wavelength bands. The reflectance of sensitive bands was used as input variables for machine learning algorithms to estimate photosynthetic capacity. The results are presented in **Table 4**. The accuracy of the model remained unaffected when using only the top eight sensitive bands instead of the full spectrum. Although there was a slight increase in the root mean square error and average error percentage compared to the full spectrum, it was not significant. The sensitive spectral band results of the SVR model in estimating *V_cmax_* and *J_max_* even exceeded the full spectrum. The results demonstrated that the estimation of photosynthetic parameters using automatically identified band reflectance was close to those of the full spectrum. The bands identified by our model yielded higher estimation of photosynthetic parameters compared to those proposed by the classic machine learning model (PLSR). Furthermore, these results validated the reliability and effectiveness of the model in identifying wavelength bands.

**Table 4:** The photosynthetic capacity estimation results of different machine learning methods for the full spectrum wavelength bands, the sensitive bands identified through Indexfindnet and the sensitive bands identified by classic machine learning model (PLSR).

Model	treat	metrics	*V_cmax_*	*J_max_*
SVR	Full	R^2^	0.43 ± 0.03	0.53 ± 0.01
	Indexfindnet Filtered		**0.45 ± 0.02**	**0.61 ± 0.01**
	PLSR Filtered		0.41 ± 0.02	0.45 ± 0.02
	Full	RMSE	20.59 ± 0.59	39.99 ± 0.22
	Indexfindnet Filtered		**20.17 ± 0.33**	**36.26 ± 0.31**
	PLSR Filtered		20.85 ± 0.65	43.00 ± 0.53
	Full	MAPE	0.28 ± 0.01	0.27 ± 0.01
	Indexfindnet Filtered		**0.27 ± 0.02**	**0.25 ± 0.01**
	PLSR Filtered		0.30 ± 0.01	0.30 ± 0.02
PLSR	Full	R^2^	0.47 ± 0.02	**0.58 ± 0.04**
	Indexfindnet Filtered		**0.49 ± 0.01**	0.51 ± 0.02
	PLSR Filtered		0.34 ± 0.02	0.43 ± 0.03
	Full	RMSE	19.71 ± 0.35	**37.81 ± 1.89**
	Indexfindnet Filtered		**19.43 ± 0.22**	40.65 ± 1.55
	PLSR Filtered		21.95 ± 0.46	43.82 ± 2.04
	Full	MAPE	0.30 ± 0.01	**0.27 ± 0.02**
	Indexfindnet Filtered		**0.29 ± 0.01**	0.31 ± 0.02
	PLSR Filtered		0.34 ± 0.02	0.32 ± 0.01

The unit of RMSE is μmol m^-2^ s^-1^. The term “Full” represents the full spectrum. “Indexfindnet Filtered” refers to the eight bands identified using Indexfindnet. “PLSR Filtered” represents the sensitive bands identified by classic machine learning model (PLSR). Specific bands are listed in **Supplementary Table S2**. Bolding indicates the best performance.

### 3.6 Performance of Indexfindnet at different spectral resolutions

**Figure 7:**
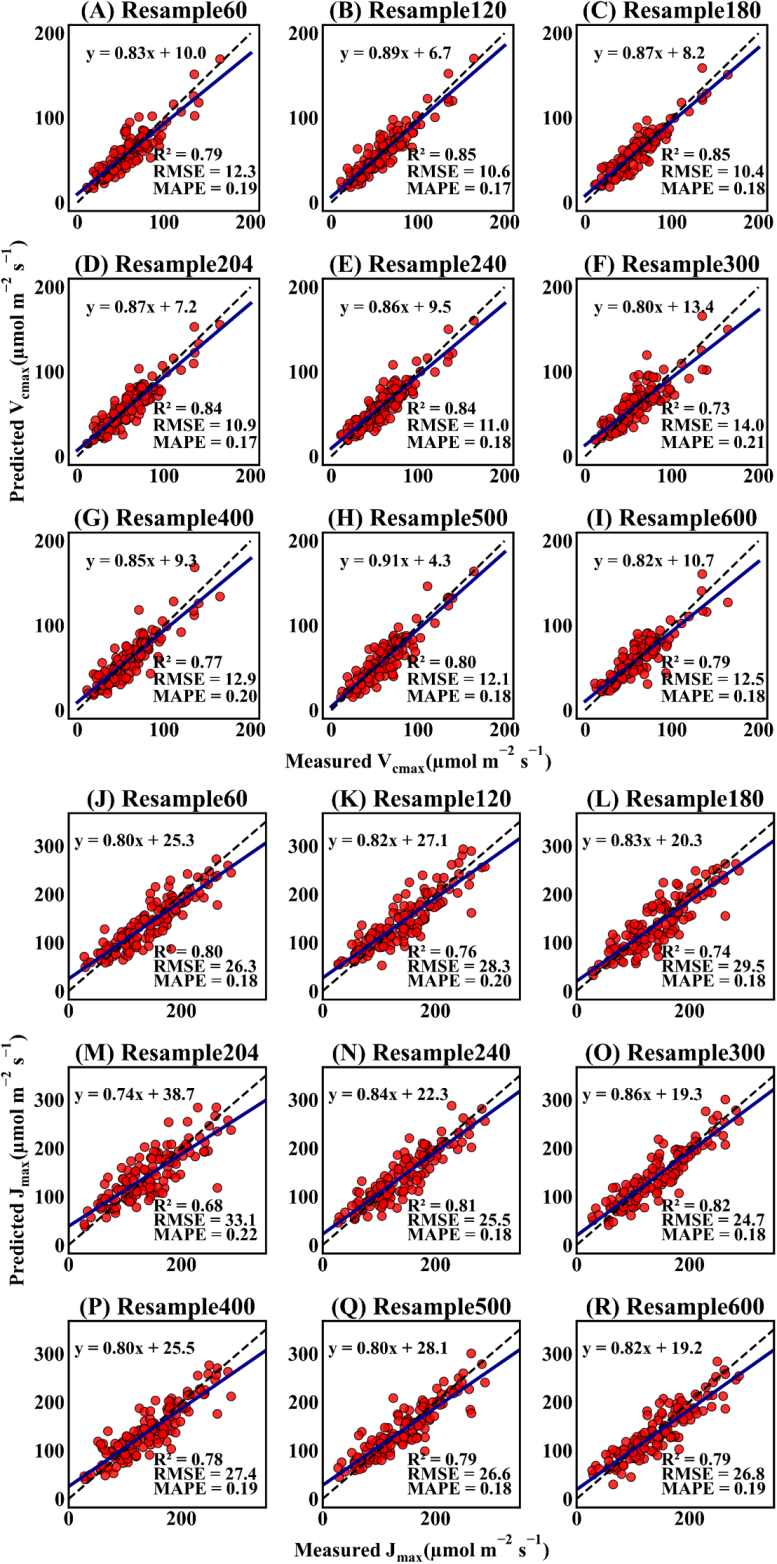
Scatter plots of estimation of the photosynthetic parameter *V_cmax_* and *J_max_* at different spectral resolutions. The “Resample 60” refers to the spectral sequence being resampled to 60 bands, which is also reflected in the naming convention used in the other subfigures. The black dashed line represents the 1:1 line at a 45-degree angle. The darkblue solid line represents the trendline of the linear fit. **(A–I)** represent the scatter plots of estimation of *V_cmax_* at different spectral resolutions, and **(J–R)** represent the scatter plots of estimation of *J_max_* at different spectral resolutions.'

To investigate the applicability of the Indexfindnet model at different spectral resolutions, the R^2^, RMSE, and MAPE were calculated to evaluate the Indexfindnet model performance (**Supplementary Table S1**). **Figure 7** demonstrates the performance of Indexfindnet in estimating the photosynthetic capacity across various spectral resolutions. Overall, there was no significant difference in the performance of the model for estimating the two photosynthetic parameters across different spectral resolutions. The spectrum with 300 bands exhibited the highest performance in estimating *V_cmax_* and *J_max_*, with an R^2^ of 0.81-0.85, RMSE of 10.5-25.6 μmol m^-2^ s^-1^, and MAPE of 17%. The spectral sequence based on 600 bands showed the poorest performance for *V_cmax_* and *J_max_*. The model showed high predictive accuracy on 1 nm resolution spectral data from previous studies (**Supplementary Figure S6**), which achieved an R² of 0.75 for *V_cmax_* and 0.79 for *J_max_*. In general, the variety in spectral resolution had minimal impact on the performance of the model.

## 4 Discussion

### 4.1 Advantage of Indexfindnet over traditional methods

Modeling photosynthetic capacity with vegetation indices showed weak ability with correlation coefficients below 0.6 (Section 3.2). This weakness may stem from challenges in finding spectral indices with optimal band combinations (Chen et al., 2022) or the limitations of linear modeling in capturing the complex nonlinear relationship between vegetation indices and photosynthetic capacity. Traditional machine learning methods like PLSR reduced spectral data to a few principal components and had weak representational capacity. The results of photosynthetic capacity estimation also performed poorly (Section 3.4). Although IndiceCNN performed well, it relied on uninterpretable features across a wide and chaotic spectrum of bands in the calculation of vegetation index formulas due to dilated convolution and pooling operators, which may result in imprecise biophysical features (Deng et al., 2024).

**Figure 8:**
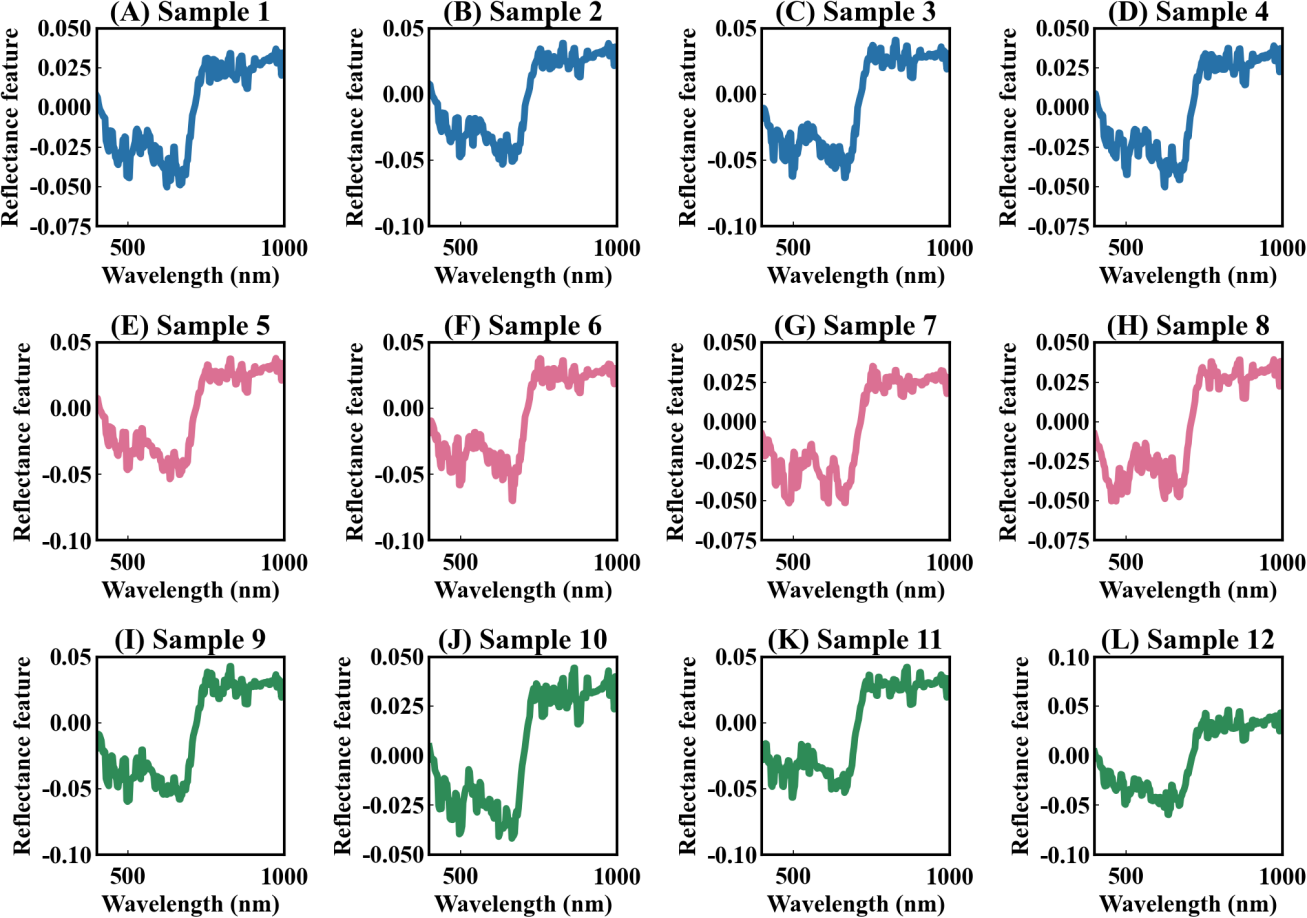
The feature maps of the Mask module. The values on the y-axis represent the weights of feature activation. **(A–L)** represent the 12 feature maps of the Mask module.

**Figure 9:**
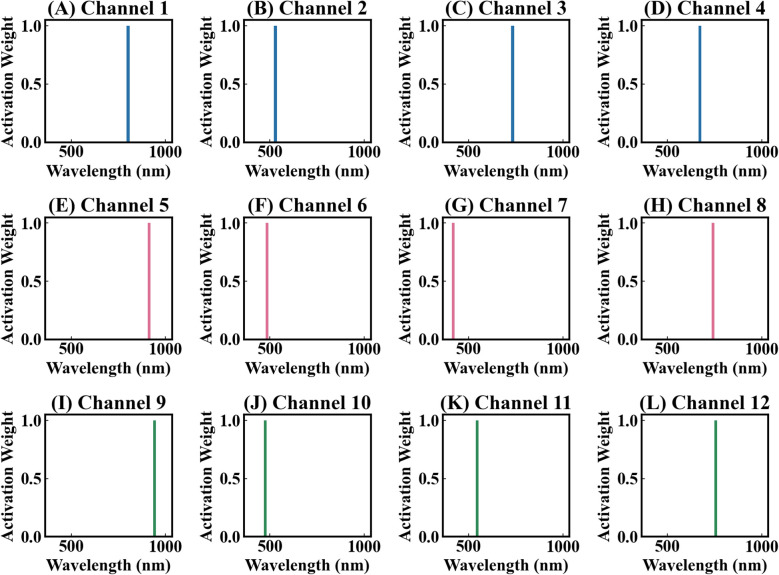
The one-hot vector found by the NonlocalBandAttention module. The index with a value of 1 in this vector represents the positions of a sensitive band. **(A–L)** represent the 12 one-hot vectors of the NonlocalBandAttention module.

The deep learning model developed in this study can effectively address these issues. Previous models that used deep learning to extract spectral features were mostly uninterpretable (Furbank et al., 2021; Wang et al., 2021b, 2022; Deng et al., 2024). In contrast, our model Indexfindnet incorporated an interpretable neural network architecture (**Figure 1**). It employed a Mask module to feature extraction. We constrained the input features of attention layer using MaskLoss. (Section 2.4.2). The feature map of the Mask module is shown in **Figure 8**. It can be seen that the reflectance characteristics of remained unchanged. The reflection troughs of blue light and red light, as well as the reflection peaks of green light, were highlighted. This was advantageous for the subsequent module NonlocalBandAttention to extract the positions of sensitive bands. A global band attention module was used to obtain a global one-hot activation vector in the NonlocalBandAttention module. **Figure 9** illustrates the global one-hot vectors obtained from different channels. These vectors served as the global alignment weight for automatic band search. They provided a deterministic band selection from global spectra on each channel rather than a probability distribution. The extracted band spectra were then fed into the vegetation index calculation module. This module integrated the biophysical features from multi sensitive bands response to photosynthetic capacity. We selected the most important index form features by gating mechanism. We can achieve precise band and vegetation index selection through this interpretable network structure.

Notably, this interpretable model exhibited superior performance in estimating photosynthetic capacity compared to traditional methods. Specifically, it demonstrated estimation accuracy above 0.8 for *V_cmax_* and *J_max_*. Among the traditional models, one-dimensional convolutional neural networks outperformed SVR and PLSR. This observation underscored the powerful feature extraction capability of deep learning. Numerous studies have successfully employed deep learning-based spectral analysis methods to predict various indicators with satisfactory results (Yu et al., 2018; Xin et al., 2020; Zhang et al., 2020; Wang et al., 2021b). These findings demonstrated the great potential of deep learning in handling high-dimensional spectral data.

### 4.2 The bands and vegetation indices searched by Indexfindnet and the underlying mechanisms of spectra response to photosynthesis

Compared to previous studies (Serbin et al., 2012; Barnes et al., 2017; Meacham-Hensold et al., 2019; Fu et al., 2020; Wang et al., 2021a), the sensitive bands identified by our model resulted in higher estimation for photosynthetic capacity (**Table 4**). This improvement is attributed to the ability of the model to target wavelengths that are directly linked to the key photosynthetic processes. These wavelengths are crucial for capturing the biochemical and structural properties of leaves, which are fundamental to understanding and estimating photosynthetic capacity. Our findings indicated that photosynthetic capacity exhibited characteristic bands predominantly in the visible regions (400-700 nm) (Section 3). Red spectra (600-700 nm) and blue spectra (400-500 nm) were the most prevalent. Green spectra (500-560 nm) and near-infrared spectra (700-1000 nm) came next. The regions identified by the model were mainly consistent with the areas where the leaves absorb (Zhang et al., 2021). PSII primarily absorbs blue light. PSI absorbs red light (Gitelson et al., 2022). Green light can efficiently drive photosynthesis once absorbed (Wolf and Blankenship, 2019; Gitelson et al., 2022). Far-red light (700-750 nm) enhances photosynthesis in synergy with shorter wavelengths (Emerson et al., 1957; Kono et al., 2020; Zhen et al., 2022). In addition to the portion absorbed by leaves, near-infrared spectra also accounted for a significant proportion. Near-infrared spectra are affected primarily by leaf structure (750-1000 nm) (Slaton et al., 2001). The *A_mes_* exposed to *IAS* has also been strongly associated with photosynthetic performance in numerous species (Sinclair et al., 1977; Longstreth et al., 1985). These structural features of leaves determine the depth into the leaf interior that visible light wavelengths can propagate and be absorbed. By focusing on biologically relevant spectral bands—such as red, blue, and NIR—the model is better aligned with the core processes of photosynthesis, enhancing its adaptability across species and environments. Moreover, the use of fewer, targeted bands makes the estimation process faster, more cost-effective.

Studies presented different sensitive band wavelengths for these specific spectral regions (Serbin et al., 2012; Barnes et al., 2017; Meacham-Hensold et al., 2019). Samples collected from different periods, regions, and species had different physical and chemical properties, such as shape and leaf thickness. As a result, the spectral response of photosynthesis also tended to be different. However, deep learning methods achieved better results than traditional machine learning methods when searching for feature wavelengths on larger datasets due to their strong feature representation ability (Serbin et al., 2012; Barnes et al., 2017; Meacham-Hensold et al., 2019; Fu et al., 2020; Wang et al., 2021a). When dealing with a large scale of spectral and photosynthetic samples, deep learning methods excelled at capturing and reconstructing more features through Indexfindnet. The selected characteristic wavelengths became more stable and accurate after multiple iterations.

The model results suggested that vegetation indices $\frac{R_{nir}+R_{g/b}}{R_{nir}\times R_{r}}$ were crucial for predicting photosynthetic capacity. It indicated that the interaction between near-infrared light and shorter-wavelength light was of significant importance for photosynthesis (Wong et al., 2020). The correlation between spectral indices and photosynthetic capacity also confirmed the model results (Section 3.2). Near-infrared spectroscopy reflects the structural characteristics of leaves, further reflecting whether the light can reach deeper parts of the leaves and be absorbed. Visible spectroscopy reflects the absorption of light by mesophyll cells. Numerous studies have demonstrated the ability and mechanisms of similar index types in relation to photosynthesis (Qian et al., 2019). The normalized difference vegetation index, proven to be a good indicator of photosynthesis, utilizes the interaction between near-infrared and red light (Gamon et al., 1995). The second most important was the double-difference vegetation indices $\frac{R_{1}-R_{2}}{R_{3}-R_{4}}$ . PRI and SIPI are both indices of this type. PRI was widely used because it represented the de-epoxidation of xanthophyll pigments and indicated an increase in zeaxanthin concentration (Garbulsky et al., 2011; Peñuelas et al., 2011; Sukhova and Sukhov, 2018). PRI is closely related to NPQ and photosynthetic efficiency (Goerner et al., 2011). The Structure Insensitive Pigment Index (SIPI) is correlated with leaf chlorophyll content (Dash and Curran, 2007). Since chlorophyll content plays a significant role in photosynthesis, derived indices based on chlorophyll content can serve as reliable indicators of photosynthetic capacity (Croft et al., 2017). By aligning with key physiological processes—such as photosystem efficiency and chlorophyll content—these indices enhance the predictive accuracy of the model, providing a more precise and biologically meaningful estimation of photosynthetic capacity.

When the full spectrum and the sensitive bands identified by Indexfindnet were used as inputs for various machine learning models, the results showed that the estimated photosynthetic capacity using the sensitive bands were either similar or even superior to those obtained using the full spectrum. These results suggested that the bands identified by Indexfindnet effectively represented the photosynthetic capacity. The slight decrease in the results was attributed to the loss of detailed information of spectral local features.

### 4.3 Impact of signal enhancement on the underlying mechanisms of spectra response to photosynthesis

The power compression transformation was widely used in processing speech spectral signal features (Li et al., 2021b). Green leaves absorb more visible light and show higher reflectance in the near-infrared region. This fact led to significant differences in reflectance values between these two parts. When training a network using criteria such as MSE, the optimization process tended to prioritize regions with larger spectral values. Because optimizing these regions resulted in a more noticeable reduction in the loss, this would lead to a blurred spectral structure in the low values region such as visible light. Therefore, applying an appropriate compression function to balance the loss disparity between different spectral regions can allow the network to capture more detailed information in the regions with weaker signals. This operation can enhance the spectral signals in the visible region and improve the quality of spectral feature extraction. Consequently, the performance of the model improved with a compression ratio below 1 and deteriorated with a compression ratio exceeding 1. This was why the accuracy of *V_cmax_* and *J_max_* estimation models can reach above 0.8 when the compression ratio is 0.6 and 0.1, respectively.

**Supplementary Figure S3** displays the sensitive bands identified by Indexfindnet under different spectra using power compression. The top eight wavelengths for *V_cmax_* were 667, 525, 905, 471, 795, 415, 935, and 750 nm. The top eight wavelengths for *J_max_* were 667, 750, 415, 471, 905, 795, 525 and 816 nm. The sensitive band distribution found by the model under mean compressed spectra was similar to the original spectrum. Because the compressed spectra did not alter the entire shape characteristics of the spectrum. Overall, the visible light range (400-700 nm) was still the most important for photosynthetic capacity. The near-infrared (700-1000 nm) light played a synergistic role but with reduced importance. The connection between near-infrared and visible light still plays a major role in predicting photosynthetic capacity (**Supplementary Table S3**).

### 4.4 Applicability of the Indexfindnet under different spectral resolutions

Our findings indicated that the proposed Indexfindnet performed well at various resolutions, both on our simulated resampled data (**Figure 7**) and previously reported experimental data (**Supplementary Figure S6**). These results increased the possibility of extending the utility of Indexfindnet to large spatial scales in handling advanced and upcoming satellite or airborne hyperspectral and multispectral data. The model achieved a slightly lower performance of estimated photosynthetic capacity when it sampled 600 bands. This was attributed to the increased difficulty for the model to determine the sensitivity of each band with more bands and the increased data redundancy.

**Supplementary Figures S4**, **S5** display the sensitive bands identified by Indexfindnet under different spectral resolutions. The model detected sensitive bands across different resolutions consistently. The distribution of sensitive bands the model identifies was more concentrated in the visible light range. There were more and higher peaks of sensitive bands in the visible light region, whether in lower or higher resolutions. The higher or lower resolution spectra obtained by resampling would affect the peak position of sensitive bands. However, the main regions remained unchanged. This made Indexfindnet a promising approach to facilitating different-scale remote sensing of photosynthetic capacity. **Supplementary Table S4** displays the vegetation indices identified by Indexfindnet at different spectral resolutions. It can be observed that the importance of the synergistic effect between near-infrared and visible light remained unchanged regardless of the changes in resolution.

### 4.5 Limitations and prospects

The newly developed Indexfindnet has shown remarkable performance in estimating photosynthetic capacity. We also verified the effectiveness of Indexfindnet to identify sensitive bands of vegetation indices within high-dimensional spectral wavelengths. We established the form of the vegetation index based on commonly used indices. Further research is needed to apply deep learning to automatically learn more complex forms of vegetation indices. Meanwhile, the applicability of the model to other species and indicators need to investigate. In this study, only one spectral preprocess method was utilized. Additional mathematical spectral treatments can be explored to enhance the accuracy of the model. This study incorporated the visible and near-infrared spectral regions. Further investigation into spectral regions that encompass the short-wave infrared portion is needed.

## 5 Conclusion

We developed an interpretable deep learning model for evaluating leaf photosynthetic capacity based on global spectral dimensional information mining. The Indexfindnet model outperformed traditional methods in estimating photosynthetic capacity. The model improved the utilization of spectral dimensional information. Visible light, especially red and blue light, was the most sensitive region identified by the model, followed by the near-infrared region. The interaction between near-infrared spectra and visible spectra was crucial for photosynthetic capacity. Signal enhancement presented an opportunity to improve the performance of deep learning using hyperspectra. Our developed model also remained stable under different resolutions. However, the performance of the model could be influenced by specific factors, such as extreme environmental conditions, poor spectral data quality and variations in spatial resolution. Additionally, its adaptability across different platforms and scalability for large datasets require further evaluation. Future research should focus on assessing the robustness of the model under diverse conditions and enhancing its efficiency for broader applicability in real-world scenarios. These advancements could provide a foundation for future research to fully explore spectral features and deep insights into the mechanisms of spectra response to photosynthesis.

## Data Availability

The datasets presented in this study can be found in online repositories. The names of the repository/repositories and accession number(s) can be found in the article/**Supplementary Material**.
