# Supplementary material for: Deep learning-enabled exploration of global spectral features for photosynthetic capacity estimation
Source: Front Plant Sci. 2025 Jan 13;15:1499875. doi: 10.3389/fpls.2024.1499875 (PMC11769944; doi:10.3389/fpls.2024.1499875)
Supplement: Supplementary file 1 [file DataSheet1.docx]

**Supplementary data**


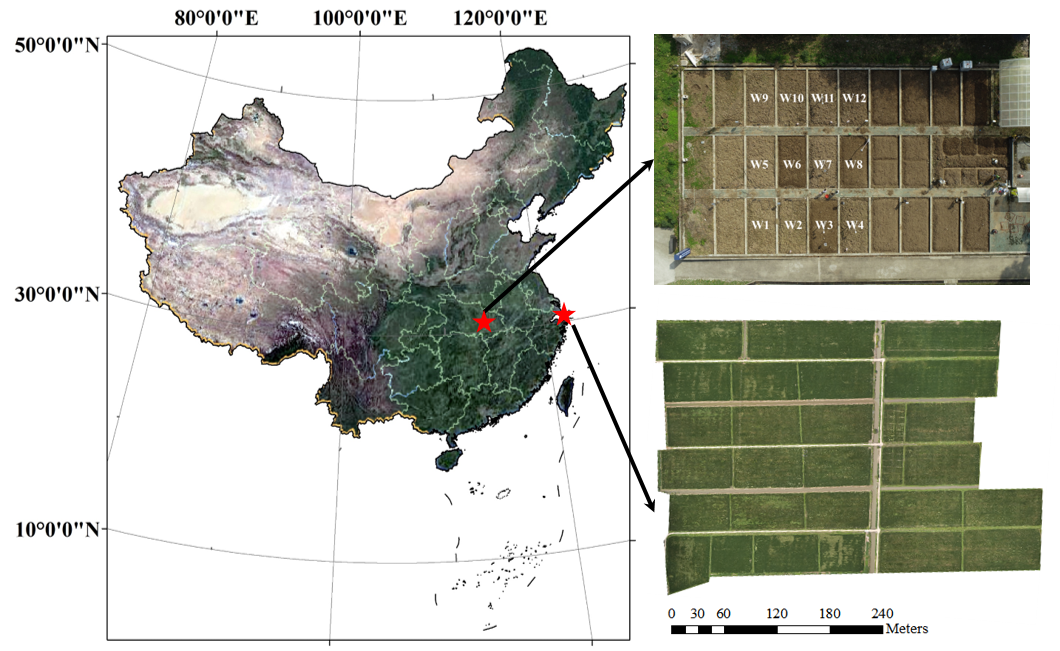


**Figure S1.** Geographic location of the experimental area. Wheat data were collected in Wuhan City, Hubei Province, China (30.54°N, 114.36°E), and rice data were collected on Hengsha Island, Chongming, Shanghai, China (31.34°N, 121.84°E).


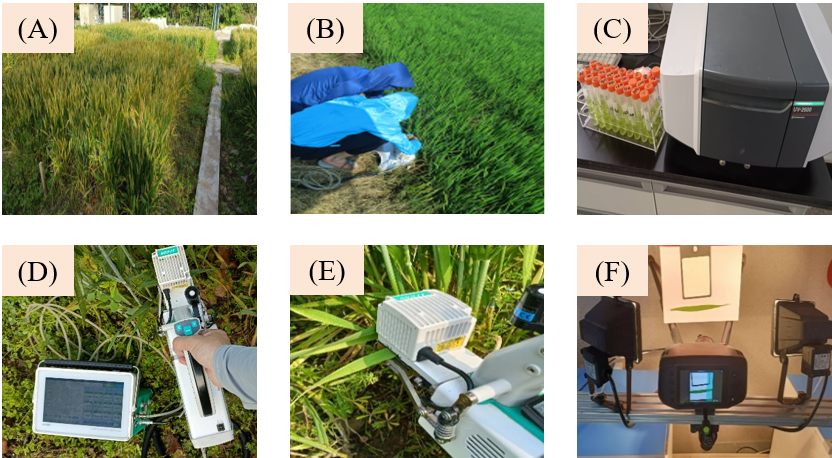


**Figure S2.** Images of the experimental data collection process. (A) Wheat experimental field. (B) Data collection in the rice field. (C) Chlorophyll content measurement. (D-E) Collection of *A*-*C_i_* curves. (F) Hyperspectral image collection.

**
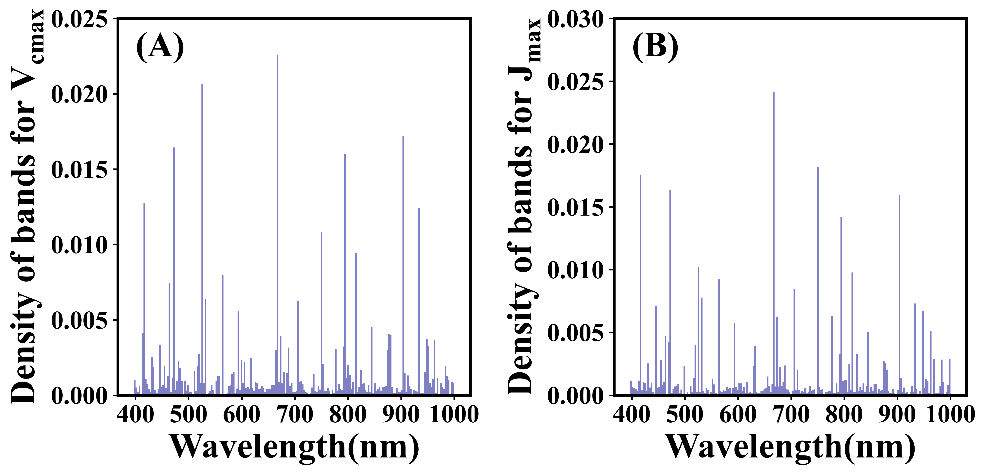
**

**Figure S3.** The distribution of sensitive bands identified by Indexfindnet under mean power compression spectra. The higher the column, the more frequently the model has detected the sensitive bands.

**
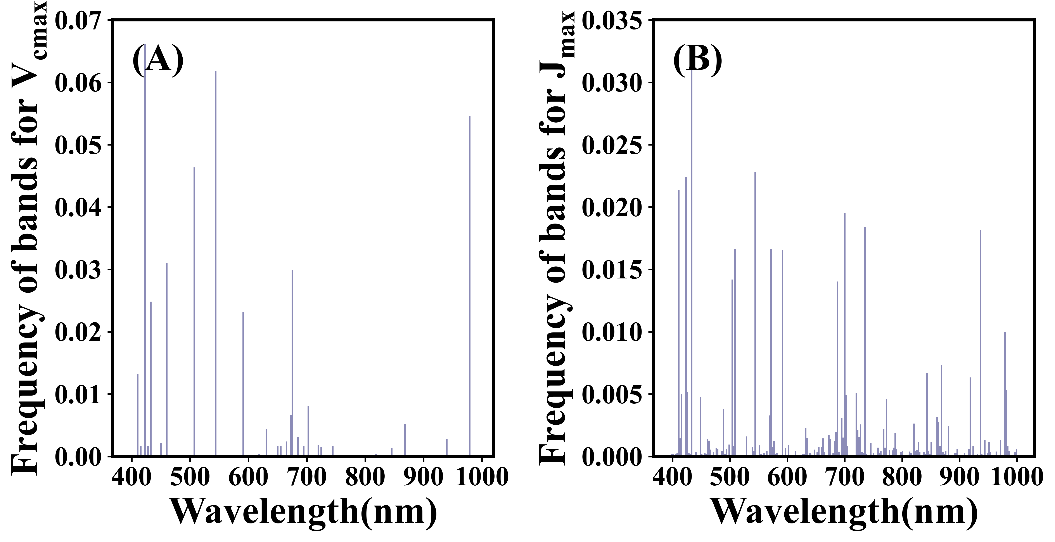
**

**Figure S4.** The distribution of sensitive bands identified by Indexfindnet under resample spectra with 240 bands. The higher the column, the more frequently the model has detected the sensitive bands.


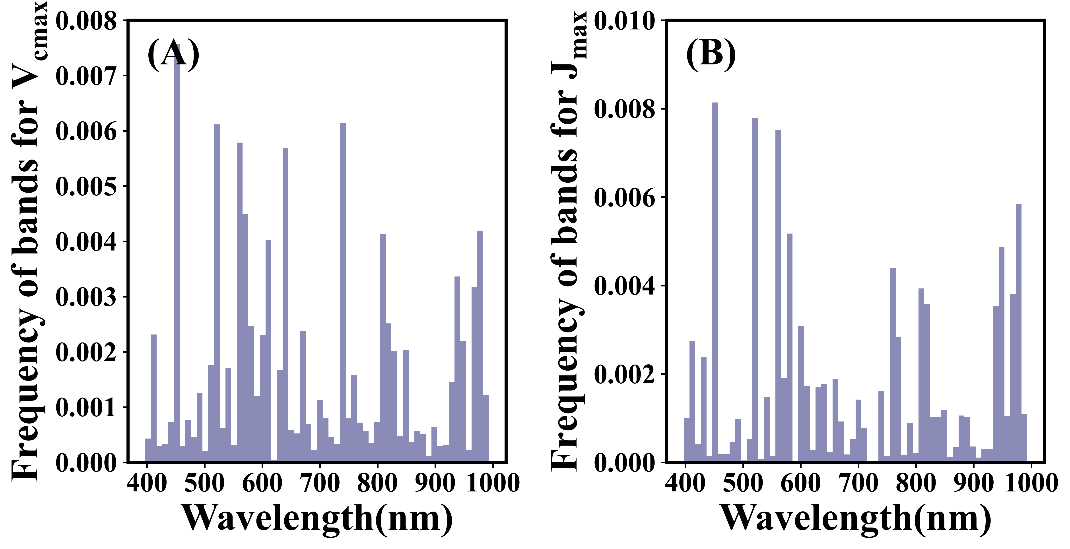


**Figure S5.** The distribution of sensitive bands identified by Indexfindnet under resample spectra with 60 bands. The higher the column, the more frequently the model has detected the sensitive bands.


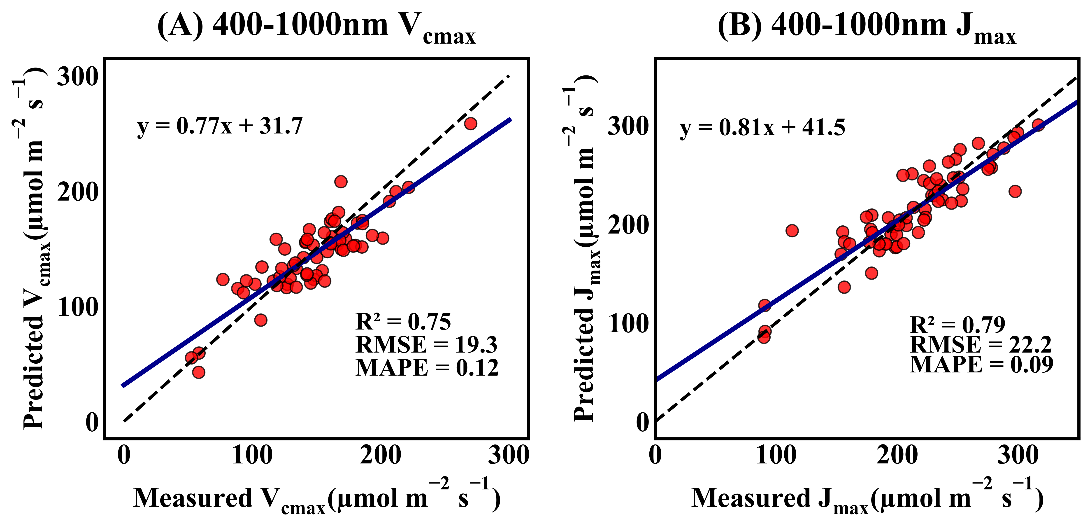

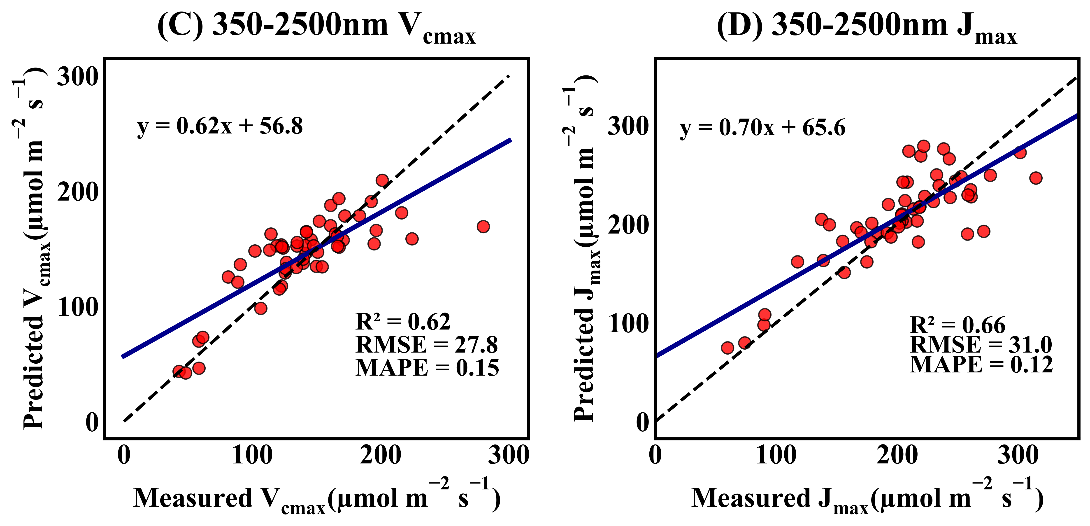


**Figure S6.** Scatter plots of estimation of the photosynthetic parameter *V_cmax_* and *J_max_* using reported actual spectral data with resolution of 1nm.

**Table S1.** Overall results of the Indexfindnet on the test set. The unit of RMSE is μmol m^-2^ s^-1^. The value of MAPE represents a percentage. For example, a MAPE with a value of 0.299 represents 29.9%. Bolding indicates the best performance. SG represents the Savitzky-Golay filtering. POC represents power compression. The number after the POC represents the compressed ratio. The number after the Resample represents the resampled bands.

| Data | | Model | *V_cmax_* | | | *J_max_* | | |
| --- | --- | --- | --- | --- | --- | --- | --- | --- |
|  |  |  | R^2^ | RMSE | MAPE | R^2^ | RMSE | MAPE |
| SG |  | SVR | 0.426 | 20.589 | 0.279 | 0.528 | 39.986 | 0.270 |
| SG |  | PLSR | 0.474 | 19.706 | 0.299 | 0.578 | 37.813 | 0.268 |
| SG |  | OneDCNN | 0.749 | 13.608 | 0.206 | 0.777 | 27.461 | 0.194 |
| SG |  | IndiceCNN | 0.834 | 11.061 | 0.176 | 0.809 | 25.415 | 0.164 |
| SG |  | Indexfindnet | 0.823 | 11.422 | 0.197 | 0.787 | 26.853 | 0.182 |
| POC | 0.1 | Indexfindnet | 0.831 | 11.157 | 0.170 | 0.811 | 25.331 | 0.168 |
| POC | 0.2 | Indexfindnet | 0.848 | 10.602 | 0.161 | 0.769 | 27.957 | 0.176 |
| POC | 0.3 | Indexfindnet | 0.852 | 10.462 | 0.159 | 0.775 | 27.609 | 0.213 |
| POC | 0.4 | Indexfindnet | 0.843 | 10.765 | 0.167 | 0.792 | 26.554 | 0.193 |
| POC | 0.5 | Indexfindnet | 0.830 | 11.220 | 0.192 | 0.762 | 28.406 | 0.206 |
| POC | 0.6 | Indexfindnet | 0.863 | 10.050 | 0.153 | 0.772 | 27.784 | 0.208 |
| POC | 0.7 | Indexfindnet | 0.840 | 10.857 | 0.174 | 0.772 | 27.769 | 0.197 |
| POC | 0.8 | Indexfindnet | 0.810 | 11.834 | 0.167 | 0.742 | 29.567 | 0.194 |
| POC | 0.9 | Indexfindnet | 0.821 | 11.487 | 0.173 | 0.777 | 27.463 | 0.195 |
| POC | 1.0 | Indexfindnet | 0.823 | 11.428 | 0.189 | 0.804 | 25.749 | 0.165 |
| POC | 1.5 | Indexfindnet | 0.805 | 12.002 | 0.181 | 0.746 | 29.316 | 0.206 |
| POC | 2.0 | Indexfindnet | 0.656 | 15.943 | 0.260 | 0.679 | 32.993 | 0.235 |
| Resample | 60 | Indexfindnet | 0.797 | 12.238 | 0.189 | 0.764 | 28.278 | 0.192 |
| Resample | 120 | Indexfindnet | 0.819 | 11.560 | 0.169 | 0.772 | 27.786 | 0.196 |
| Resample | 180 | Indexfindnet | 0.827 | 11.315 | 0.177 | 0.793 | 26.515 | 0.191 |
| Resample | 204 | Indexfindnet | 0.804 | 12.028 | 0.186 | 0.787 | 26.853 | 0.182 |
| Resample | 240 | Indexfindnet | 0.836 | 11.009 | 0.174 | 0.789 | 26.724 | 0.190 |
| Resample | 300 | Indexfindnet | 0.849 | 10.547 | 0.169 | 0.807 | 25.571 | 0.167 |
| Resample | 400 | Indexfindnet | 0.799 | 12.191 | 0.179 | 0.798 | 26.178 | 0.185 |
| Resample | 500 | Indexfindnet | 0.788 | 12.514 | 0.184 | 0.773 | 27.737 | 0.210 |
| Resample | 600 | Indexfindnet | 0.773 | 12.935 | 0.195 | 0.727 | 30.393 | 0.209 |

**Table S2.** The sensitive bands response to photosynthetic parameters identified by PLSR. Sensitive bands are identified by sorting the absolute values of the coefficients from the PLSR model.

| Sensitive bands *V_cmax_* | Sensitive bands *J_max_* | Order |
| --- | --- | --- |
| 560 nm | 446 nm | 1 |
| 423 nm | 405 nm | 2 |
| 397 nm | 408 nm | 3 |
| 411 nm | 519 nm | 4 |
| 437 nm | 945 nm | 5 |
| 501 nm | 648 nm | 6 |
| 738 nm | 481 nm | 7 |
| 795 nm | 420 nm | 8 |

**Table S3.** Spectral indices searched out by the Indexfindnet in power compression spectra. Index1 refers to the most frequently searched index by the model, Index2, Index3, Index4, and so on in a similar manner. Index refers to the universally summarized indices derived. *R_nir_* represents the reflectance of near-infrared bands. *R_g/b_* represents the reflectance of green or blue bands. *R_r_* represents the reflectance of red bands.

| trait | Index1 | Index2 | Index3 | Index4 | **Index** |
| --- | --- | --- | --- | --- | --- |
| *V_cmax_* | $\frac{R_{471}+R_{525}}{R_{905}\times R_{667}}$ | $\frac{R_{471}+R_{525}}{R_{935}\times R_{667}}$ | $\frac{R_{795}+R_{525}}{R_{905}\times R_{750}}$ | $\frac{R_{795}+R_{525}}{R_{905}\times R_{667}}$ | $\frac{\boldsymbol{R}_{\boldsymbol{nir/b}}\boldsymbol{+}\boldsymbol{R}_{\boldsymbol{g}}}{\boldsymbol{R}_{\boldsymbol{nir}}\boldsymbol{\times}\boldsymbol{R}_{\boldsymbol{r}}}$ |
| *J_max_* | $\frac{R_{471}+R_{415}}{R_{905}\times R_{750}}$ | $\frac{R_{471}+R_{415}}{R_{935}\times R_{750}}$ | $\frac{R_{471}+R_{415}}{R_{905}\times R_{667}}$ | $\frac{R_{795}+R_{525}}{R_{905}\times R_{750}}$ | $\frac{\boldsymbol{R}_{\boldsymbol{nir/b}}\boldsymbol{+}\boldsymbol{R}_{\boldsymbol{g/b}}}{\boldsymbol{R}_{\boldsymbol{nir}}\boldsymbol{\times}\boldsymbol{R}_{\boldsymbol{r}}}$ |

**Table S4.** Spectral indices searched out by the Indexfindnet under resample spectra with 60 or 240 bands. Index1 refers to the most frequently searched index by the model, Index2, Index3, Index4, and so on in a similar manner. Index refers to the universally summarized indices derived. *R_nir_* represents the reflectance of near-infrared bands. *R_g/b_* represents the reflectance of green or blue bands. *R_r_* represents the reflectance of red bands.

| bands | trait | Index1 | Index2 | Index3 | Index4 | **Index** |
| --- | --- | --- | --- | --- | --- | --- |
| 60 | *V_cmax_* | $\frac{R_{609}+R_{740}}{R_{953}\times R_{559}}$ | $\frac{R_{609}+R_{740}}{R_{983}\times R_{560}}$ | $\frac{R_{831}+R_{740}}{R_{950}\times R_{560}}$ | $\frac{R_{609}+R_{740}}{R_{760}\times R_{821}}$ | $\frac{\boldsymbol{R}_{\boldsymbol{nir/r}}\boldsymbol{+}\boldsymbol{R}_{\boldsymbol{r}}}{\boldsymbol{R}_{\boldsymbol{nir}}\boldsymbol{\times}\boldsymbol{R}_{\boldsymbol{b}}}$ |
|  | *J_max_* | $\frac{R_{942}+R_{427}}{R_{760}\times R_{821}}$ | $\frac{R_{942}+R_{427}}{R_{760}\times R_{559}}$ | $\frac{R_{982}+R_{427}}{R_{760}\times R_{559}}$ | $\frac{R_{942}+R_{427}}{R_{982}\times R_{559}}$ | $\frac{\boldsymbol{R}_{\boldsymbol{nir}}\boldsymbol{+}\boldsymbol{R}_{\boldsymbol{b}}}{\boldsymbol{R}_{\boldsymbol{nir}}\boldsymbol{\times}\boldsymbol{R}_{\boldsymbol{b}}}$ |
| 240 | *V_cmax_* | $\frac{R_{980}+R_{422}}{R_{675}\times R_{543}}$ | $\frac{R_{980}+R_{410}}{R_{422}\times R_{900}}$ | $\frac{R_{980}+R_{591}}{R_{508}\times R_{897}}$ | $\frac{R_{427}+R_{422}}{R_{675}\times R_{897}}$ | $\frac{\boldsymbol{R}_{\boldsymbol{nir}}\boldsymbol{+}\boldsymbol{R}_{\boldsymbol{b/g}}}{{\boldsymbol{R}_{\boldsymbol{b/r}}\boldsymbol{\times R}}_{\boldsymbol{nir}}}$ |
|  | *J_max_* | $\frac{R_{980}+R_{422}}{R_{737}\times R_{543}}$ | $\frac{R_{980}+R_{937}}{R_{737}\times R_{543}}$ | $\frac{R_{427}+R_{937}}{R_{737}\times R_{543}}$ | $\frac{R_{427}+R_{591}}{R_{737}\times R_{543}}$ | $\frac{\boldsymbol{R}_{\boldsymbol{nir/b}}\boldsymbol{+}\boldsymbol{R}_{\boldsymbol{nir/b}}}{\boldsymbol{R}_{\boldsymbol{nir}}\boldsymbol{\times}\boldsymbol{R}_{\boldsymbol{b}}}$ |

**References**

**Furbank RT, Silva-Perez V, Evans JR, Condon AG, Estavillo GM, He W, Newman S, Poiré R, Hall A, He Z**. 2021. Wheat physiology predictor: predicting physiological traits in wheat from hyperspectral reflectance measurements using deep learning. Plant Methods **17**, 1–15.

**Deng X, Zhang Z, Hu X, Li J, Li S, Su C, Du S, Shi L**. 2024. Estimation of photosynthetic parameters from hyperspectral images using optimal deep learning architecture. Computers and Electronics in Agriculture **216**, 108540.
